# Comparison of a recombinant endonuclease III protein and its synthetic peptides in ELISA for the diagnosis of tegumentary leishmaniasis using human serum and urine samples: A preliminary study

**DOI:** 10.1371/journal.pntd.0014440

**Published:** 2026-06-15

**Authors:** Raquel S. B. Câmara, Dóris M. Abrão, Daniela P. Lage, Camila S. Freitas, Ana L. Silva, Mariana M. Cardoso, Nathália C. Galvani, Maíza M. Rodrigues, Breno L. Pimenta, Bárbara P. N. Assis, Ana T. Chaves, Unaí Tupinambás, Manoel O. da Costa Rocha, Miguel A. Chávez-Fumagalli, Ricardo A. Machado-de-Ávila, Denise U. Gonçalves, Isabela A. G. Pereira, Eduardo A. F. Coelho, Myron Christodoulides

**Affiliations:** 1 Programa de Pós-Graduação em Ciências da Saúde: Infectologia e Medicina Tropical, Faculdade de Medicina, Universidade Federal de Minas Gerais, Belo Horizonte, Minas Gerais, Brazil; 2 Programa de Pós-Graduação em Ciências da Saúde, Universidade do Extremo Sul Catarinense, Criciúma, Santa Catarina, Brazil; 3 Hospital Eduardo de Menezes, Fundação Hospitalar do Estado de Minas Gerais, Belo Horizonte, Minas Gerais, Brazil; 4 Computational Biology and Chemistry Research Group, Vicerrectorado de Investigación, Universidad Católica de Santa María, Umacollo, Arequipa, Peru; 5 Departamento de Patologia Clínica, COLTEC, Universidade Federal de Minas Gerais, Belo Horizonte, Minas Gerais, Brazil; 6 Neisseria Research Group, Molecular Microbiology, School of Clinical and Experimental Sciences, University of Southampton Faculty of Medicine, Southampton General Hospital, Southampton, England; Fundação Oswaldo Cruz: Fundacao Oswaldo Cruz, BRAZIL

## Abstract

**Background:**

The laboratory diagnosis of tegumentary leishmaniasis (TL) remains challenging, mainly because of the variable sensitivity and specificity of tests used. Moreover, biological samples are typically collected through invasive procedures. In this pilot study, the recombinant *Leishmania* endonuclease III (rENDO) protein and two specific B-cell epitopes predicted from its amino acid sequence were evaluated as diagnostic antigens for TL, by using paired serum and urine samples from patients.

**Methodology/Principal findings:**

rENDO protein, two synthetic peptides and a Soluble *Leishmania* Antigen (SLA) extract as comparator, were used to develop an ELISA that was tested with a controlled panel of paired urine and serum samples from 175 patients. Results showed that the serum-based ELISA reported sensitivity of 100% for rENDO, Peptide 1, and Peptide 2, and of 85.0% for SLA. Specificity values were of 100%, 100% and 96.6% for the protein and peptides, and 67.3% for SLA, respectively. For the urine-based ELISA, sensitivity was also of 100% for rENDO, Peptide 1, and Peptide 2, and of 90.6% for SLA. Specificity values were 100%, 100% and 96.5%, for the protein and peptides, and 91.5% for SLA, respectively. The antibody response was compared with a commercial kit, and results showed a kappa index higher than 0.90 for rENDO using both serum and urine samples, whereas the kit showed values below 0.80. Additionally, a one-point longitudinal study for antibody response monitoring in treated ML patients revealed a drop in rENDO-specific IgG levels of nearly 50% after six months.

**Conclusions:**

Preliminary data suggest that the ELISA using rENDO and its derived peptides demonstrated good sensitivity and specificity for identifying TL cases, and the rENDO-based assay could potentially have a role in monitoring the treatment of TL patients.

## Author summary

Diagnosing tegumentary leishmaniasis (TL) is often difficult because current laboratory tests vary in accuracy and usually require invasive sample collection, such as drawing blood or taking tissue from skin lesions. In this preliminary study, a laboratory approach that uses a *Leishmania* protein called recombinant endonuclease III (rENDO), along with two small pieces of this protein (called peptides), was evaluated to see whether they could reliably detect TL. Importantly, not only blood samples were tested, but also urine, which is much easier and non-invasive for collection. A total of 175 paired patient samples were available for the study. In ELISA, the rENDO protein and both peptides correctly identified all TL patients, while a traditional antigen (called SLA) performed less well. When urine samples were tested, rENDO and the peptides again detected all TL cases, showing 100% accuracy for sensitivity, while SLA detected most but not all patients. Performance was compared with a commercial diagnostic kit: rENDO performed better than the commercial test when using either blood or urine, showing a stronger agreement with patients’ disease status. In patients receiving treatment, the levels of antibodies against rENDO dropped six months after therapy. This suggests that rENDO might be useful also to follow the therapeutic regimen and contribute to indicating patient cure.

## Introduction

Leishmaniasis is a neglected tropical disease (NTD) complex caused by infection with protozoa of the genus *Leishmania*. The parasite is transmitted through the bite of infected female phlebotomine sandflies, and the disease is endemic in over 90 countries across Asia, Africa, the Americas, the Mediterranean, and parts of Southern Europe. Across these continents, 380 million people are exposed to the risk of infection, resulting in an estimated 1.5 to 2.0 million new cases and 70,000 deaths annually [1]. The disease is presented in two major clinical forms, known as tegumentary leishmaniasis (TL) and visceral leishmaniasis (VL). There are three clinical presentations within TL: 1) the cutaneous form (CL) is localized and characterized by one or more ulcerated skin lesions at the site of the sandfly bite; 2) the mucosal form (ML) involves destructive lesions of the mucous membranes, especially of the nose, mouth, and throat; and 3) the cutaneous-diffuse form (CDL) is marked by multiple non-ulcerative nodules spread across the body, and is often associated with a poor host immune response to *Leishmania* [2]. Noticeably, TL can cure spontaneously, but symptoms can be exacerbated, and disfiguring mucosal lesions result in patient morbidity [3].

TL is caused by several species of *Leishmania,* including *Leishmania braziliensis, L. major, L. aethiopica, L. amazonensis, L. guyanensis, L. panamensis* and *L. mexicana* [4]. In Brazil, TL is caused mainly by *L. braziliensis*, and a wide spectrum of clinical manifestations is observed from single CL lesions to mucosal scars characteristic of ML [5] The current treatment for TL, regardless of the parasite species causing the disease, is based on the use of pentavalent antimonials, amphotericin B and its liposomal formulations, paramomycin, amongst others: however, the toxicity, high cost and/or increasing parasite resistance to the drugs have hampered their efficacy [6,7]. Prompt treatment also requires rapid diagnosis, which is essentially based on clinical evaluation coupled with laboratory tests [8]. The gold standard for diagnosis is direct visualization of the parasite from skin lesion scrapings, isolation of the parasite in culture in a sterile environment and inoculation in animals, i.e., essentially fulfilling Kock’s postulates. However, this gold standard has variable sensitivity of 60–95% in cultures and smears [8]. Molecular diagnostic methods, e.g., polymerase chain reaction (PCR) and Loop-Mediated Isothermal Amplification (LAMP) have higher sensitivity, but their use is limited due to their high cost and the need for expensive laboratory equipment manned by trained professionals [9,10].

Immunological diagnosis of TL has the significant patient-centered advantage over the parasitological tests of less invasive sample procurement, whether blood or urine [11]. However, sensitivity and/or specificity of tests is variable due to the scarcity of serum antileishmanial antibodies, especially from CL patients and by cross-reactivity found in samples from patients developing ML [12,13]. However, blood collection can still be challenging in remote areas with restricted access to health care, or from patients with phobia, or from children [8]. Urine is an alternative and facile sample to collect, and indeed has been tested as an analyte for diagnosing Chikungunya virus infection [14], tuberculosis [15] and leprosy [16], leptospirosis [17], neurocysticercosis [18], toxoplasmosis [19] and VL [20,21]. Our recent work to develop serodiagnostic assays for leishmaniasis has focused on identifying parasite antigens with diagnostic potential, following immunoproteomics of sera from patients with disease. Several antigens have been identified, including endonuclease III (ENDO), which is a cytoplasmic glycosylase enzyme involved in *Leishmania* DNA repair and replication, and has homologues present in other parasites such as *Trypanosoma cruzi* [22] and *Plasmodium falciparum* [23]. In a recent study, the *Leishmania* ENDO III was identified in *L. infantum* protein extracts by sera from VL patients, but not by sera from patients with Chagas disease or from healthy individuals. Following on, we produced a recombinant version of ENDO (rENDO) and demonstrated that it had high sensitivity and specificity for VL diagnosis [24]. Since there is considerable amino acid sequence homology of ENDO amongst different *Leishmania* spp., for the current pilot study, we tested the hypothesis that rENDO and two specific B-cell epitopes identified *in silico* could be diagnostic antigens for TL in enzyme-linked immunoassays (ELISA) using a controlled paired urine and serum panel as biological samples. A parasite antigen preparation and a commercial kit were used as comparators with the samples. Our main goal was to develop a potential new antigenic candidate as a diagnostic and/or prognostic marker for TL for future studies. The data presented herein are preliminary and the serum and urine panels used in the experiments were pre-existing archival samples, well-controlled and known. Such factors may mask the true diagnostic accuracy obtained, and when new samples are tested, whether derived from different locations or countries or collected in the field, they may result in reduced sensitivity and specificity values, as will be reported in our work.

## Methods

### Ethics statement

All participants signed the informed consent form on inclusion. The study was approved by the Ethics Committee of the Federal University of Minas Gerais (UFMG, Belo Horizonte, Minas Gerais, Brazil), lodged under protocol number CAAE-32343114.9.0000.5149. The study complied with ethical standards outlined in Declaration of Helsinki.

### The parasite and preparation of the soluble *Leishmania* protein extract (SLA)

Stationary promastigotes of *L. braziliensis* strain MHOM/BR/1975/M2904 were grown at 24ºC in Schneider’s medium (Sigma-Aldrich, USA), supplemented with 20% (v/v) inactivated fetal bovine serum (FBS, Sigma), 20 mM L-glutamine, 200 U/mL penicillin, and 100 µg/mL streptomycin, pH 7.4. SLA was prepared from 10^9^ stationary promastigotes as described previously [25]. Briefly, parasites (10^9^ cells) were washed three times in sterile phosphate-buffered saline (PBS, pH 7.4) and subjected to six cycles of freezing in liquid nitrogen (-196ºC) and thawing in water (+37^o^C), followed by sonication (Ultrasonic processor, GEX600, with five cycles of 30 seconds at 38 MHz) and centrifugation (9,000 x g for 30 min at 4^o^C). The supernatant containing SLA was stored at -70^o^C, until use.

### Study population, disease diagnoses and sample collection

The study followed a single-center, case-control, phase-I diagnostic accuracy design using controlled archival samples available already in our laboratory. A total of 175 samples were used in the experiments. Urine and serum from patients with CL (n = 25) and ML (n = 30), multibacillary leprosy (n = 20), Chagas disease (n = 25), malaria (n = 10), and HIV infection (n = 20) were evaluated in ELISA. In addition, paired samples were collected from healthy subjects living in endemic region of TL (n = 45; Belo Horizonte, Minas Gerais, Brazil), and they did not present sign or symptom of disease or from other infectious diseases. Urine (20 mL) was collected early in the morning into a tube containing preservative (0.1% (w/v) sodium azide (NaN_3_; catalog 71289, Sigma-Aldrich, USA) and was stored at 4°C. Blood (10 mL) was obtained through venipuncture using a sterile vacuum collection tube with clot activator and separator gel. Tubes were centrifuged (3,500 x rpm for 15 min at 4°C), and the serum was separated and stored at -70°C.

TL cases, represented by CL and ML patients, were diagnosed by expert clinicians and with laboratory assays of skin and/or tissue biopsies. Giemsa-stained smears from lesion fragments and Polymerase Chain Reaction (PCR) to detect *L. braziliensis* kDNA were used as diagnostic tools [26,27]. Briefly, the parasite kDNA was extracted from samples through the QIAamp DNA Blood Mini Kit (Qiagen, MD, USA). It was used to amplify a specific *L. braziliensis* kDNA region (150 base pairs). The following primers were used in the reactions: 5´-GGGKAGGGGCGTTCTSCGAA-3´ (*Forward*) and 5´-SSSWCTATWTTACACCAACCCC-3´ (*Reverse*). *Leishmania* kDNA was also extracted from a reference *L. braziliensis* strain (MHOM/BR/2002/LPC-RPV) and used as positive control, and sterile ultrapure water was used as negative control. The following reagents were used in the reactions: 2 mM MgCl_2_, 200 µM dNTPs, 0.6 µM of each primer (Sigma-Aldrich, USA), 1 UI Taq DNA polymerase with specific buffer (Invitrogen, USA), and 20 ng DNA templates. The program applied was: (step 1) 94ºC for 10 min; (step 2) 60ºC for 30 seconds; (step 3) 72ºC for 30 seconds; (step 4) 94ºC for 30 seconds and go to step 2 for 42 times; (step 5) 72ºC for 10 min [28]. All CL and ML patients presented positive PCR results. None of them had any previous history of other infectious diseases and did not receive any treatment at the time of urine and serum collection. Chagas disease was diagnosed by hemoculture, Chagatest recombinant ELISA kit and/or Chagatest hemmaglutination inhibition (Wiener lab., Rosario, Argentina). Multibacillary leprosy was diagnosed by clinical evaluation and histopathological examination of lesion biopsy. Malaria was diagnosed by clinical evaluation and positive test though microscopy from Giemsa-stained blood drops. HIV infection was confirmed by clinical assay of the patients, laboratory tests counting CD4^+^ and CD8^+^ T cell subtypes, and a serological test for detection of anti-HIV IgG antibodies.

### Prediction of ENDO B-cell epitopes and peptide synthesis

We have used well-standardized protocols of bioinformatics developed by our group to predict B cell epitopes from antigenic proteins. Initially, the FASTA sequence of ENDO (CAC9452939.1) protein was obtained from GenBank. It was aligned using the Clustal Omega program [29], where the epitope regions were compared with similar sequences obtained through Blast-P. IEDB server (www.iedb.org) was used to identify the most accessible amino acids in the protein primary structure, by using the parameters of window size of 14 and threshold of 1.0. B-cell epitopes were predicted using the ABCpred server (www.imtech.res.in/raghava/abcpred/) [30], which can predict linear B cell epitopes based on fixed-length patterns through an artificial neural network, with the parameters of Threshold of 0.85, Window length of 16, and Overlapping filter ON. PepCalc server (www.pepcalc.com) was used to characterize the predicted B-cell epitopes. Peptides were synthetized as described previously [31], lyophilized and stored at -70^o^C until use, whereupon they were reconstituted in milli-Q water to a final concentration of 1.0 mg/mL.

### Production of recombinant (r)ENDO protein

The gene encoding ENDO was cloned and expressed in *E. coli* as described previously [24]. Purification of the recombinant protein was done on a HisTrap HP affinity column connected to an AKTA system (GE Healthcare, USA), Amicon ultra15 centrifugal filters 10,000 NMWL (Millipore, Germany), a Superdex 200 gel-filtration column (GE Healthcare Life Sciences, USA) and an agarose-polymyxin column (Sigma-Aldrich, USA), as described previously [24]. Protein concentration was estimated by using the BCA protein assay reagent kit (Thermo Scientific, Waltham, USA) according to manufacturer’s instructions.

### Serum-based and urine-based ELISA

Previous titration curves were established to evaluate the appropriate ENDO/SLA concentration and serum/urine dilution and antibody-conjugate dilution to be used for the assays. ELISA was done in 96-well microtiter immunoassay plates (Jetbiofil, Belo Horizonte). For the serum-based ELISA, wells were coated with rENDO, Peptide 1, Peptide 2, and SLA at 1.0, 0.5, 0.5 and 1.0 µg per well, respectively. For the urine-based ELISA, wells were coated with rENDO, Peptide 1, Peptide 2, and SLA at 1.0, 0.5, 1.0, and 1.0 µg per well, respectively. All antigens were diluted in 100 µL of 50 mM carbonate coating buffer, pH 9.6 and stored at 4°C for 16 h. After cold storage, free binding sites were blocked with 200 µL of PBS, pH 7.4 plus Tween 20 (PBST) containing 1% (w/v) bovine serum albumin (BSA) for 1 h at 37°C. Plate wells were washed five times with PBST and sera were tested at a 1/200 dilution in PBST (100 µL per well) and urine undiluted (100 µL per well) with incubation for 1 h at 37°C. Plate wells were washed six times with PBST and for the serum-based ELISA, they were incubated with anti-human IgG horseradish-peroxidase conjugated antibody (catalog A18811, Invitrogen, USA), at dilutions of 1/40,000, 1/20,000, 1/20,000, and 1/10,000 in PBST for rENDO, Peptide 1, Peptide 2 and SLA, respectively. For the urine-based ELISA, a 1/5000 dilution of conjugate was used. The plates were incubated for 1 h at 37°C and then washed six times with PBST. Colorimetric reactions were developed by incubation with 100 μL per well of 3,3´,5,5’ tetramethylbenzidine (Scienco, Brasil) for 30 min in the dark for the serum-based ELISA and 20 min for the urine-based ELISA. All reactions were stopped by adding 50 µL of 2 N H_2_SO_4_ per well. The optical density (OD) values were read in an ELISA microplate reader (Molecular Devices, Spectra Max Plus, Canada) at λ450 nm. All urine and serum samples were codified prior to use, and the study was conducted in a blind manner by our group. Samples were evaluated using the same reagents (lots, dilutions, etc) to reduce possible experimental variations. Two different ELISA procedures were done by independent people using the same serum and urine samples, and results obtained were similar between them. Finally, a Bland-Altman plot analysis showed no significant difference in OD values between the duplicates performed.

### Comparative analysis using a commercial kit

The Detect Rapid Test (InBios International Inc., USA), which is an immunochromatographic test for the qualitative detection of antibodies against the conserved k39 protein from *Leishmania* spp., was used according to the manufacturer’s instruction. The kit was used as an antigen control against urine and serum samples of the TL patients to evaluate the performance of the rENDO protein.

### Serological follow-up

Paired urine and serum samples were collected from 15 ML patients who were different from the TL (CL and ML) patients previously evaluated in the ELISA trials (n = 30). Thus, paired samples from 15 previously unstudied patients were collected before treatment and six months after treatment with pentavalent antimonials (Sanofi Aventis Farmacêutica Ltda., Suzano, São Paulo, Brazil). Samples were individually tested by ELISA to evaluate the specific antibody response. As described above, two different procedures were independently performed by separate operators using the same serum and urine samples, and similar results were obtained between them.

### Statistical analyses

Data were entered into Microsoft Excel (version 10.0) spreadsheets and analyzed with GraphPad Prism version 10.0.2 for Windows (GraphPad Software, USA, www.graphpad.com). The cut-off values were calculated through Receiver Operating Characteristic (ROC) curves, which were plotted with the values from samples from *Leishmania*-infected patients versus those from healthy and cross-reactive disease (CRD) groups. The accuracy was evaluated according to the Area Under the Curve (AUC) relative to the ROC curve (95%CI) and Youden (Y) index. Tables of Contingency and Fisher’s exact test (*P* < 0.05) were used to calculate sensitivity (Se), specificity (Sp), likelihood ratio (LR), positive (PPV) and negative (NPV) predictive values, with a confidence interval at 95% (95%CI).

## Results

### B-cell epitope prediction by bioinformatics

**Fig 1:**
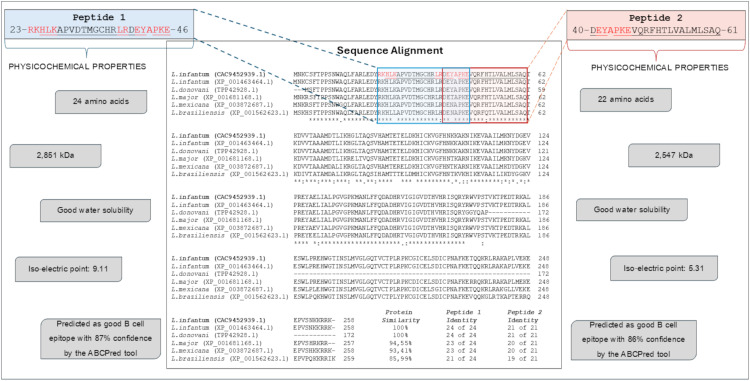
Analysis of the endonuclease III amino acid sequences in *Leishmania* species and prediction of B-cell epitopes. Multiple sequence alignment of the endonuclease III protein (CAC9452939.1) was done for distinct *Leishmania* species by using BLASTp and Clustal Omega servers. The region spanning amino acid residues 23 to 60 was strongly predicted with epitope properties, which was based on bioinformatics assays using IEDB Surface Accessibility and ABCPred tools. Residues predicted as surface-accessible are shown in bold, while those predicted as B-cell epitopes are underlined. Peptide 1 (23-46 residues; in blue) and Peptide 2 (40-61 residues; in red) were designed from the region and the physicochemical characteristics of the peptides are shown along either side. The overall sequence similarity among *Leishmania* species and the identity of the peptides across species is reported below the sequence alignment.

Multiple sequence alignments of the ENDO sequence from *Leishmania* species causing TL and VL were made through the BLASTp and Clustal Omega servers, and B-cell epitopes were predicted with ABCPred. The region between amino acids 23–60 was strongly predicted as containing an epitope (Fig 1), and two peptides were designed and synthesized from this region. These peptides showed good sequence homology between the evaluated parasite species. Peptide 1 (23-RKHLKAPVDTMGCHRLRDEYAPKE-46; shown in blue) had 24 amino acids, molecular weight (Mr) of 2,851 daltons and an isoelectric point (IEP) of 9.11, and Peptide 2 (40-DEYAPKEVQRFHTLVALMLSA-61; shown in red) had 22 amino acids, Mr of 2,547 daltons and IEP of 5.31. ABCPred predicted Peptide 1 and 2 as having 87.0% and 86.0% probability of being antigenic epitopes, respectively. Pepcalc predicted good water solubility for both peptides. The bioinformatics data for BLAST, IEDB Emini surface accessibility prediction, ABCpred and Clustal alignments are provided in S1 Data.

### Preliminary application of rENDO protein, synthetic peptides and SLA *L. (V.) braziliensis* for the diagnosis of TL

**Fig 2:**
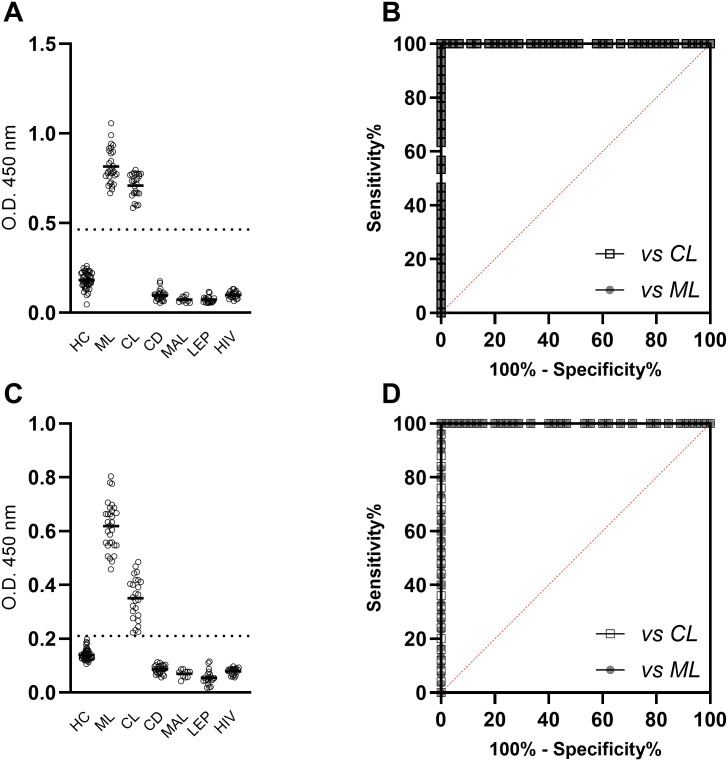
Recombinant ENDO protein evaluated for the diagnosis of tegumentary leishmaniasis. Experiments were performed using serum (A) and urine (C) samples from patients with CL (n = 25) and ML (n = 30), multibacillary leprosy (n = 20), Chagas disease (n = 25), malaria (n = 10), and HIV-infected (n = 20). In addition, paired samples from healthy subjects living in endemic regions of disease (n = 45) were also used. The dotted lines indicate the cut-off values, while solid lines indicate the mean OD values. ROC curves were constructed and results are shown by using serum (B) and urine (D) as biological samples.

We developed an *in-house* ELISA using rENDO as antigen and tested it against paired urine and serum samples from patients with TL, as well as against other cross-reactive diseases (CRD) and samples from healthy adults (HC). As shown in Fig 2, antibodies in both serum (Fig 2A) and urine (Fig 2C) from CL and ML patients (combined as TL) reacted with rENDO antigen, with OD values above the cut-off value. By contrast, antibodies in serum and urine from healthy adults and CRD patients, i.e., with confirmed Chagas disease, malaria, leprosy and HIV infection, had OD values below the cut-off values. ROC curves were constructed for serum (Fig 2B) and urine (Fig 2D) and the data showed promising diagnostic performance, with sensitivity, specificity, PPV, NPV and Youden index values of 100%, 100%, 100%, 100%, and 1.0, respectively, for TL when compared with the other groups (Table 1).

**Table 1:** Diagnostic evaluation of rENDO, peptides and SLA for TL using serum and urine samples.

Serum								
Antigen	Comparison	Fisher’s exact test	Se (95% CI)	Sp (95% CI)	PPV (95% CI)	NPV (95% CI)	LR	Y
rENDO	TL vs HC	<0.0001	100 (93.5-100)	100 (93.5-100)	100 (93.5-100)	100 (93.5-100)	NC	1
rENDO	TL vs CRD	<0.0001	100 (93.5-100)	100 (93.5-100)	100 (93.5-100)	100 (93.5-100)	NC	1
Peptide 1	TL vs HC	<0.0001	100 (93.2-100)	95.7 (85.8-99.2)	96.4 (87.7-99.4)	100 (92.1-100)	23.50	0.95
Peptide 1	TL vs CRD	<0.0001	100 (93.2-100)	97.4 (91.0-99.5)	96.4 (87.7-99.4)	100 (95.1-100)	38.50	0.97
Peptide 2	TL vs HC	<0.0001	100 (93.5-100)	100 (93.5-100)	100 (93.5-100)	100 (93.5-100)	NC	1
Peptide 2	TL vs CRD	<0.0001	100 (93.5-100)	100 (93.5-100)	100 (93.5-100)	100 (93.5-100)	NC	1
SLA	TL vs HC	<0.0001	100 (88.7-100)	64.3 (52.6-74.5)	54.6 (41.5-67.0)	100 (92.1-100)	2.8	0.64
SLA	TL vs CRD	<0.0001	69.8 (54.9-81.4)	71.3 (61.0-79.7)	54.6 (41.5-67.0)	82.7 (72.6-89.6)	2.43	0.4
**Urine**								
**Antigen**	**Comparison**	**Fisher’s exact test**	**Se (95% CI)**	**Sp (95% CI)**	**PPV (95% CI)**	**NPV (95% CI)**	**LR**	**Y**
rENDO	TL vs HC	<0.0001	100 (93.5-100)	100 (93.5-100)	100 (93.5-100)	100 (93.5-100)	NC	1
rENDO	TL vs CRD	<0.0001	100 (93.5-100)	100 (93.5-100)	100 (93.5-100)	100 (93.5-100)	NC	1
Peptide 1	TL vs HC	<0.0001	100 (93.5-100)	100 (93.5-100)	100 (93.5-100)	100 (93.5-100)	NC	1
Peptide 1	TL vs CRD	<0.0001	100 (93.5-100)	100 (93.5-100)	100 (93.5-100)	100 (93.5-100)	NC	1
Peptide 2	TL vs HC	<0.0001	100 (93.5-100)	100 (93.5-100)	100 (93.5-100)	100 (93.5-100)	NC	1
Peptide 2	TL vs CRD	<0.0001	100 (93.5-100)	100 (93.5-100)	100 (93.5-100)	100 (93.5-100)	NC	1
SLA	TL vs HC	<0.0001	100 (92.9-100)	90 (78.6-95.7)	90.91(80.4-96.1)	100 (92.1-100)	10.00	0.90
SLA	TL vs CRD	<0.0001	83.33 (72.0-90.7)	92.86 (84.3-96.9)	90.91 (80.4-96.1)	86.67 (77.2-92.6)	11.67	0.76

Table 1. Diagnostic evaluation of rENDO, Peptide 1, Peptide 2 and SLA for TL. Serum and urine samples were collected from cutaneous (CL; n = 20) and mucosal (ML; n = 25) leishmaniasis patients, which were called tegumentary leishmaniasis (TL) group; as well as from patients with CL (n = 25) and ML (n = 30), multibacillary leprosy (n = 20), Chagas disease (n = 25), malaria (n = 10), and HIV-infected (n = 20).Paired samples from healthy subjects living in endemic region of disease (n = 45) were also used. Patient groups were classified as cross-reactive diseases (CRD) group. The paired samples were used in ELISA experiments against the rENDO protein, Peptide 1 and Peptide 2 and SLA, when individual optical density values obtained were used to construct ROC curves. The diagnostic assay was performed by calculating sensitivity (Se), specificity (Sp), confidence intervals (95%CI), negative (NPV) and positive (PPV) predictive values, likelihood ratio (LR), and Youden index (Y). NC, not calculated.

**Fig 3:**
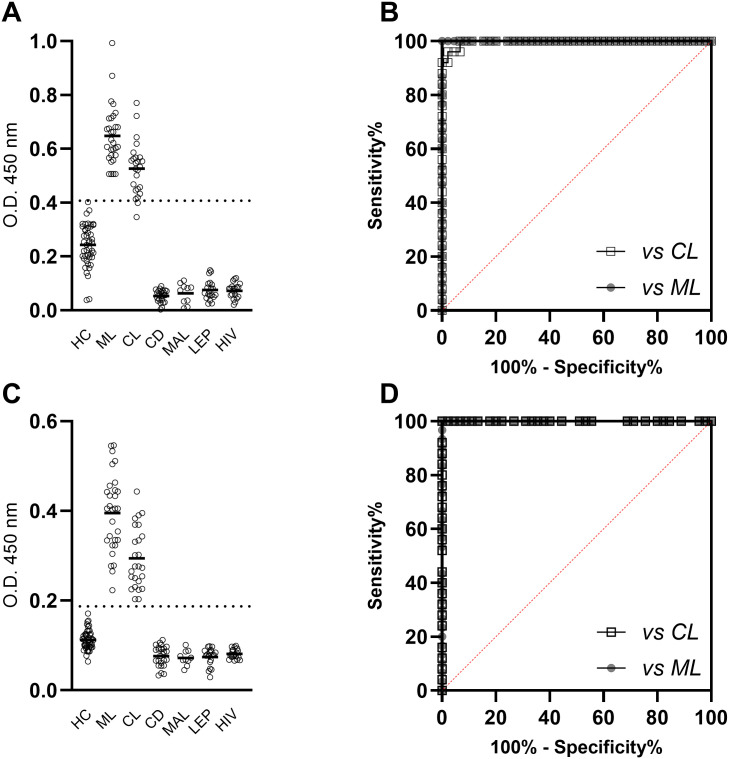
Peptide 1-based ELISA for the diagnosis of tegumentary leishmaniasis. Experiments were conducted using serum (A) and urine (C) samples from patients with CL (n = 25) and ML (n = 30), multibacillary leprosy (n = 20), Chagas disease (n = 25), malaria (n = 10), and HIV-infected (n = 20). In addition, paired samples from healthy subjects living in endemic regions of disease (n = 45) were also used. The dotted lines indicate the cut-off values, while solid lines indicate the mean OD values. ROC curves were constructed and results are shown by using serum (B) and urine (D) as biological samples.

**Fig 4:**
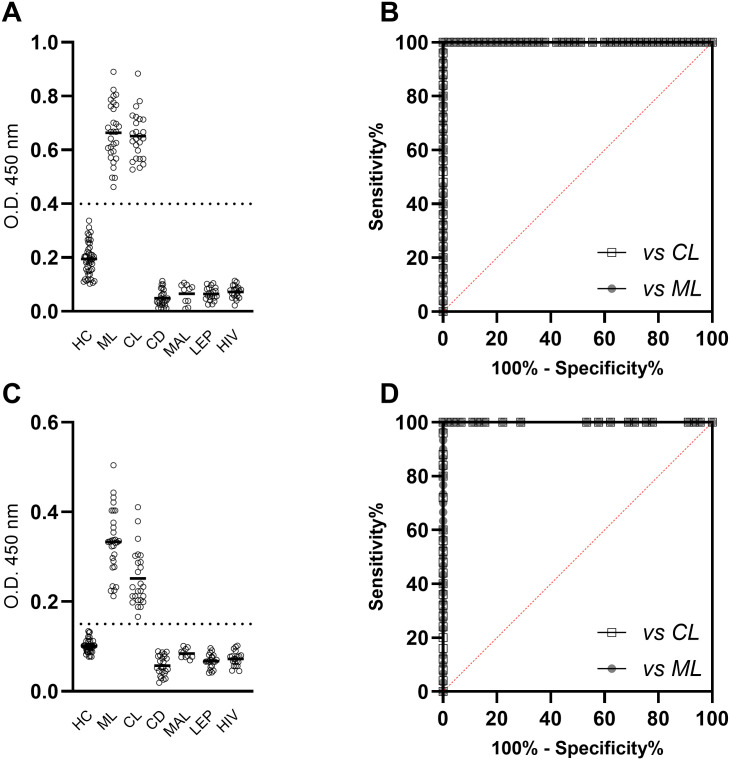
Peptide 2-based ELISA for the diagnosis of tegumentary leishmaniasis. ELISA was developed using serum (A) and urine (C) samples from patients with CL (n = 25) and ML (n = 30), multibacillary leprosy (n = 20), Chagas disease (n = 25), malaria (n = 10), and HIV-infected (n = 20). In addition, paired samples from healthy subjects living in endemic regions of disease (n = 45) were also used. The dotted lines indicate the cut-off values, while solid lines indicate the mean OD values. ROC curves were constructed and results are shown by using serum (B) and urine (D) as biological samples.

Next, ELISA experiments were done with urine and serum samples tested on Peptide 1 (Fig 3) and Peptide 2 (Fig 4). Results showed also a promising diagnostic evaluation for both antigens, with CL and ML patients showing individual OD values above the cut-off, when urine and serum were tested against Peptide 1 (Fig 3) and Peptide 2 (Fig 4). On the other hand, samples from HC and CRD patients presented OD values below the cut-off. ROC curves were constructed, and the serum-based ELISA showed sensitivity, specificity, PPV, NPV, and Youden index values for Peptide 1 of 100%, 96.6%, 96.4%, 100%, and 0.96, respectively. The urine-based ELISA for Peptide 1 showed values of 100%, 100%, 100%, 100%, and 1.0, respectively. For Peptide 2, all parameters were 100%, 100%, 100%, 100%, and 1.0 in both assays (Table 1).

**Fig 5:**
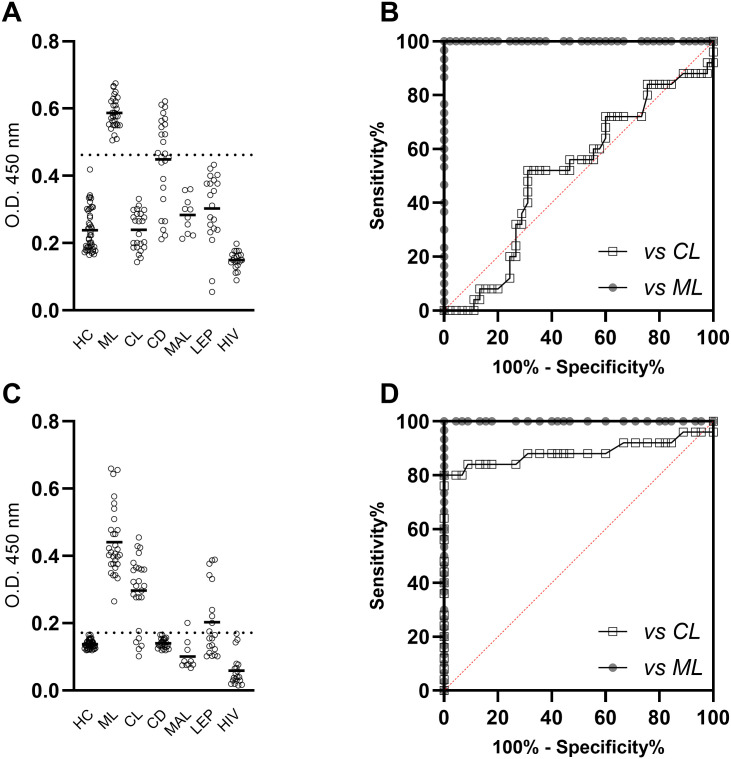
SLA *L. (V.) braziliensis* used as antigen for diagnosis of tegumentary leishmaniasis. Experiments were conducted by using serum (A) and urine (C) samples from patients with CL (n = 25) and ML (n = 30), multibacillary leprosy (n = 20), Chagas disease (n = 25), malaria (n = 10), and HIV-infected (n = 20). In addition, paired samples from healthy subjects living in endemic regions of disease (n = 45) were also used. The dotted lines indicate the cut-off values, while solid lines indicate the mean OD values. ROC curves were constructed and results are shown by using serum (B) and urine (D) as biological samples.

SLA *L. (V.) braziliensis* was used as a comparator antigen in the ELISA experiments (Fig 5), and results showed that samples from ML patients presented OD values above the cut-off, confirming positive reactivity with this this multi-antigenic preparation (Fig 5). CL patients presented lower humoral reactivity against SLA, and cross-reactivity was found with samples from Chagas disease (serum) and leprosy (urine) patients. ROC curves were constructed, and serum-based ELISA showed values of sensitivity, specificity, PPV, NPV and Youden index in the order of 85.0%, 67.3%, 54.6%, 91.0%, and 0.63, respectively; while urine-based ELISA showed values of 90.6%, 91.5%, 90.9%, 91.5%, and 0.83, respectively (Table 1).

The accuracy data from the ROC curves were used to evaluate the performance of the antigens for identifying ML and CL cases (Table 2).

**Table 2:** Accuracy data from the ROC analysis for identification of tegumentary leishmaniasis. Receiver Operating Characteristic (ROC) curves, which were plotted with the values from samples from *Leishmania*-infected patients versus those from healthy and cross-reactive disease (CRD) groups. The accuracy was evaluated according to the Area Under the Curve (AUC) relative to the ROC curve (95%CI).

Antigen	Disease		Serum			Urine	
		AUC	95%CI	*p*-value	AUC	95%CI	*p*-value
rENDO	To identify ML	1.0	1.0-1.0	<0.0001	1.0	1.0-1.0	<0.0001
rENDO	To identify CL	1.0	1.0-1.0	<0.0001	1.0	1.0-1.0	<0.0001
Peptide 1	To identify ML	1.0	1.0-1.0	<0.0001	1.0	1.0-1.0	<0.0001
Peptide 1	To identify CL	0.99	0.98-1.0	<0.0001	1.0	1.0-1.0	<0.0001
Peptide 2	To identify ML	1.0	1.0-1.0	<0.0001	1.0	1.0-1.0	<0.0001
Peptide 2	To identify CL	1.0	1.0-1.0	<0.0001	1.0	1.0-1.0	<0.0001
SLA	To identify ML	1.0	1.0-1.0	<0.0001	1.0	1.0-1.0	<0.0001
SLA	To identify CL	0.51	0.37-0.65	0.86	0.88	0.77-0.99	<0.0001

### Comparison with a commercial kit and antibody follow-up in treated patients

We also evaluated the antibody response from CL and ML patients with a commercial kit, to compare reactivity with the rENDO protein (Table 3). rENDO-specific reactivity was found in 53 and 51 of 55 urine and serum samples of the TL patients, respectively, with calculated kappa index of 0.97 and 0.91, respectively. Using the commercial kit, 43 and 36 of 55 urine and serum samples from TL patients, respectively, were reactive, and the calculated kappa index was of 0.71 and 0.65, respectively.

**Table 3:** Comparison of rENDO and a commercial kit for the diagnosis of TL. The diagnostic response from only TL patients against rENDO was compared with a commercial kit.

1. Performance of the antigens on tegumentary leishmaniasis (TL) using urine (55 samples from TL patients)		
	False-negative	True-positive
rENDO	2 (4%)	53 (96%)
Kit	12 (22%)	43 (78%)
2. Performance of the antigens on tegumentary leishmaniasis (TL) using serum (55 samples from TL patients)		
	False-negative	True-positive
rENDO	4 (7%)	51 (93%)
Kit	19 (35%)	36 (65%)

**Fig 6:**
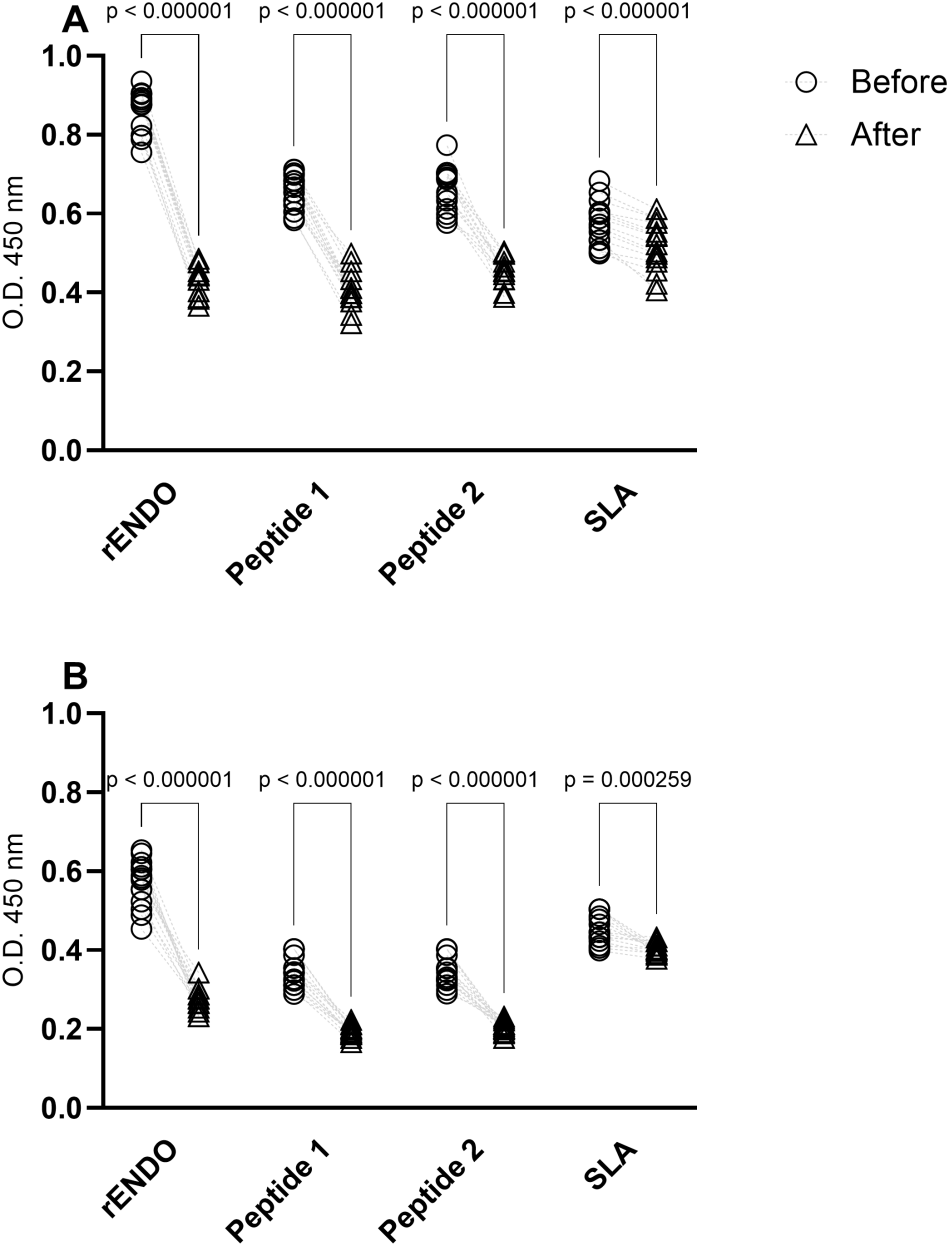
Comparison of antibody titers in TL patients at different treatment time points. Sera (A) and urine (B) samples from patients with tegumentary leishmaniasis (n = 15) were collected before and 6 months after treatment, and levels of anti-ENDO, anti-Peptide1, anti-Peptide2, or anti-SLA IgG antibodies were measured. Circles and triangles indicate the individual optical density (OD) values for each sample against antigens, before and after treatment, respectively.

We also examined the antibody response in treated patients. For this, urine and serum samples had been collected previously from another 15 ML patients, before and after treatment, and the humoral reactivity against rENDO, Peptide 1, Peptide 2 and SLA was evaluated. Results showed that antibodies declined ~40–50% after treatment, when rENDO, Peptide 1 and Peptide 2 were used in the plates. SLA tested as antigen showed a similar antibody reactivity with samples collected before and after patient treatment. Urine-based ELISA showed IgG reductions in the order of 54.4%, 43.3%, 39.4%, and 10.5%, when rENDO, Peptide 1, Peptide 2 and SLA were used, whereas with the serum-based ELISA, IgG reductions were 49.7%, 37.6%, 29.6%, and 9.7%, respectively (Fig 6).

## Discussion

TL is a NTD complex capable of causing significant psychological, physical, and social impacts, largely due to scarring and mutilating lesions that contribute to patient morbidity [32]. Improvements in diagnosis are needed to mitigate these effects, and a laboratory test should be sensitive, specific, and require only minimally invasive sample collection [7]. Therefore, identifying novel and more sensitive and specific antigens, as well as alternative and less invasive analytes, represents a promising strategy for improving TL diagnosis. In this preliminary study, we evaluated the performance in ELISA of the rENDO protein, and two synthetic peptides derived from its amino acid sequence using both serum and urine as samples for TL. Our rationale was based on the use of synthetic peptides and recombinant proteins in studies aiming to diagnose TL, with the preliminary findings being encouraging [11,33]. Our recent works have also highlighted the potential of using patient urine reacting with recombinant proteins and synthetic peptides as an alternative approach for TL diagnosis [34,35]. In our current study, the key findings were a satisfactory performance, although preliminary, of the rENDO-based and peptide-based ELISA, which was consistent with other studies in which such antigens were evaluated for the diagnosis of leishmaniasis. The serum-based ELISA showed good sensitivity and specificity for diagnosing both CL and ML cases. The good performance to detect CL patients is relevant, since these individuals usually produce low levels of anti-*Leishmania* antibodies compared to ML patients, which often results in reduced sensitivity in laboratory trials [36–38]. In general, CL and ML exhibit distinct clinical manifestations in patients, differing in location, progression, and severity. CL is typically confined to the skin, presenting as one or more painless ulcers with indurated borders at exposed sites, and it often follows a chronic but self-limited course with spontaneous healing and scar formation. In contrast, ML involves the mucous membranes of the upper respiratory tract, particularly the nasal and oropharyngeal regions, and usually develops months to years after an initial cutaneous lesion due to parasite dissemination. Unlike the localized and relatively benign nature of cutaneous disease, mucosal involvement is progressive and destructive, characterized by chronic inflammation, ulceration, and significant tissue loss, leading to symptoms such as nasal obstruction, epistaxis, dysphonia, and dysphagia. Patients with CL tend to have a higher parasite load with moderate immune response, whereas ML is associated with low parasite burden but an exaggerated inflammatory response, contributing to severe tissue damage and the need for prompt systemic treatment. Such factors provide clinicians with the means to differentiate between CL and ML in patients [39–42]. In this context, data presented herein can be considered satisfactory, since the ENDO antigen was able to identify both cases of both diseases, when humoral reactivity was evaluated. These findings aid in the safe and precise diagnosis of leishmaniasis, in contrast to other infectious diseases with overlapping symptoms resembling TL. In addition, the use of urine, compared to serum, indicated that it can be an alternative biological analyte to diagnose the disease [20,43–47]. However, limitations include the low number of paired samples used herein and, therefore, the work should be considered as a single-center, case-control, phase-I diagnostic study in which controlled urine and serum samples were applied in the ELISA experiments.

Other studies have also reported similar results using urine in ELISA for the diagnosis of leishmaniasis. For example, Ghosh et al. [48] used urine from 87 VL patients and 81 healthy individuals, which were tested against the rK28, rK39, and rKRP42 proteins. The authors reported sensitivity of 95.4%, 94.3%, and 90.8%, respectively, and specificity of 96.3%, 97.5%, and 96.2%, respectively, to diagnose VL. The authors suggested that urine-based antibody detection ELISA may serve as a useful non-invasive diagnostic tool in remote areas. Similarly, Câmara et al. [49] evaluated a parasite cytoplasmic protein, such as ENDO studied here, namely recombinant eukaryotic initiation factor 5a (eIF5a) protein, and a predicted B-cell epitope, as antigens for TL diagnosis. The authors found that the eIF5a protein and its synthetic peptide showed sensitivities of 44.0% and 29.3% and specificities of 100% and 99.0%, respectively, in serum-based ELISA, and sensitivities of 100% and 48.0% and specificities of 99.1% and 98.9%, respectively, in urine-based ELISA. They concluded that eIF5a could be a suitable antigen for urine-based diagnostic testing for TL. Other studies have also used urine to diagnose distinct diseases. For example, Ramos et al. [50] evaluated a recombinant chimeric protein composed of B-cell epitopes from the spike (S) and nucleocapsid (N) proteins of the SARS-CoV-2 virus in an in-house ELISA, using both urine and serum samples from COVID-19 patients. Their results showed 83.7% sensitivity and 100% specificity when serum samples were tested, and 91.1% sensitivity and 100% specificity when urine was used as the analyte. The authors suggested that this chimeric antigen could be considered a diagnostic marker for SARS-CoV-2 infection, with either serum or urine applicable in the assay. Chungkanchana et al. [51] evaluated urine for detecting parasite-specific IgG in an ELISA for the diagnosis of strongyloidiasis. They compared samples from healthy controls and from patients with low-negative, high-negative, and low-positive IgG levels, and found significantly elevated IgG levels in the low-positive group, yielding 100% positive results. The authors concluded that urine-based ELISA could be a potentially useful tool for the diagnosis of strongyloidiasis. In another parasite study, Toribio et al. [52] developed a dipstick assay to detect *Taenia solium* antigens in urine samples from 30 neurocysticercosis patients and 10 healthy subjects. The urine-based assay detected positivity in 29 of 30 cases and was negative in all control samples. The authors considered their work a proof-of-concept for a functional urine antigen point-of-care assay that may aid in the diagnosis of neurocysticercosis.

Accurate diagnosis of leishmaniasis is essential towards efficient control of this NTD, but it presents problems related to the sensitivity and/or specificity of the tests [53]. In fact, antibody cross-reactions can occur mainly with other Trypanosomatides antigens, and the use of more refined antigens, such as synthetic peptides, could limit this cross-reactivity [54]. Additionally, synthetic peptides are easier to produce and standardize. The data from our present study are encouraging, since the use of synthetic peptides shows good sensitivity and specificity like the use of SLA. This finding can be explained by the fact that amino acid sequences able to cause cross-reactivity with other antibodies are eliminated and thus it may be possible to select and construct only peptides specific to detect TL or VL, or both diseases [55].

Urinary antibodies have been used in the diagnosis of diseases, since they are found in detectable levels in infected patients [56,57]. Diseases such as filariasis [58], schistosomiasis [59], strongyloidiasis [60], dengue [61] and VL [46,62], among others, have been diagnosed in previous studies by using patient urine in laboratory analyses. However, for TL, few studies have reported using urine as the biological analyte [35,47,49]. The advantages of urine as an analyte include no invasive collection by the patient and urinary antibodies have been shown to be stable for a long period of time. Recently, Câmara et al. [35] performed a stability test using 18 urine samples from TL patients, and no significant variation in the ELISA optical density values was found in relation to of the levels of anti-LiHyV antibodies, when samples were stored for six months at 4^o^C. Similar findings were shown by others regarding the stability of urinary antibodies generated during different diseases [50,63,64]. In our study, a pilot stability assay showed also that anti-ENDO antibodies remained at similar levels even after three months storage at 4^o^C. Thus, the promising stability data reflect the possible long-term use of urine as a biological analyte for the immunodiagnosis of leishmaniasis.

It is well established that conserved cytoplasmic proteins of *Leishmania* spp., including kinesins, histones, and heat shock proteins, among others, exhibit strong seroreactivity in the detection of leishmaniasis. However, these antigens frequently display cross-reactivity with sera from patients suffering from other infectious diseases, such as Chagas disease, leprosy, and malaria [65,66]. Parasitic diseases are often co-endemic, particularly in underdeveloped regions, which facilitates the development of such cross-reactive immune responses. Several studies have demonstrated that parasite proteins share significant amino acid sequence homology with orthologues from other trypanosomatids, thereby contributing to cross-reactivity observed in immunological diagnostic assays [67–69].

ENDO is a cytoplasmic protein found in Trypanosomatides, which in *Leishmania* is related to the DNA repair and parasite replication [70]. This antigen is a known antileishmanial target, in which drugs, such as meglumine antimoniate act to revert the *Leishmania* viability [71]. ENDO also plays an immunogenic role, since it was identified in parasite antigenic extracts in an immunoproteomics study performed in *L. infantum* with reactive antibodies from VL [24]. In the same study, ENDO was evaluated also as a serodiagnostic candidate for VL [24]. These observations suggest that cytoplasmic proteins can perform important biological functions, including roles in parasite survival and virulence, and therefore may serve as promising biological candidates for leishmaniasis studies [72]. Recombinant *Leishmania* antigens have been extensively evaluated as diagnostic tools for leishmaniasis, and cytoplasmic proteins have also been explored for this purpose. Antigens such as the A2 protein [73,74], enolase [75], and heat shock proteins [76,77], among others, have been cloned, and their recombinant forms have been used in ELISA and other diagnostic platforms to detect leishmaniasis. These antigens are primarily metabolic and structural proteins associated with parasite survival and virulence, as well as stress-related proteins and molecular chaperones. Notably, the presence of cytoplasmic proteins in the amastigote stage allows closer interaction with the host immune system, particularly due to the reduced abundance of membrane-associated antigens in this life stage of *Leishmania* [78]. ENDO, another cytoplasmic parasite protein, has previously been recognized by antibodies in patients with active visceral leishmaniasis (VL) [24], and in the present study, its recombinant form was also shown to exhibit antigenicity suitable for the identification of leishmaniasis cases.

Thus far, our study has shown the potential of rENDO as a diagnostic antigen for TL. However, to improve the sensitivity and specificity of such antigen-based immunodiagnostic tests, synthetic peptides have been studied as replacements for parasite extracts such as SLA and recombinant proteins. The rationale for using synthetic peptides is that they can enhance the accuracy and robustness of diagnostic tests by eliminating uninformative or undesirable epitopes present in native antigens, which are often responsible for background or nonspecific reactions [79]. Also, their chemical synthesis obviates the need to handle living organisms and leads to molecules of high purity and consistent batch-to-batch reproducibility [80]. Indeed, when reviewing the use of recombinant proteins as antigens—including proteins such as KMP-11, LiP2, K39, K26, A2, KE16, LiHyD, LiHyG, among others—these have been used in ELISA assays with variable sensitivity and specificity. Such variability arises from factors that include the nature/structure of the recombinant antigen, the serological panel analysed and the study area [12–14,49]. Serological tests have not been used for TL diagnosis, because of these issues of low sensitivity and variable specificity [11,26]. In this context, and to increase the degree of antibody detection, synthetic peptides containing specific B-cell epitopes could be developed and used as alternatives to recombinant protein antigens. In our study, both Peptide 1 and Peptide 2 showed a commendable diagnostic performance for detecting TL. In fact, although combined predictions using the ABCPred and IEDB tools identified a continuous region of 39 amino acids within the ENDO protein sequence, which could account for the presence of a single epitopic region, we chose to synthesize the peptides separately. This decision was guided by desirable characteristics of the effective epitopes, such as structural flexibility and short length (≤20–25 amino acids), both of which could hypothetically enhance immunoreactivity. In addition, from a practical perspective, the synthesis of shorter peptides was faster, more cost-effective, and yielded products of high purity [31]. Conversely, a peptide of 39 amino acids would be more prone to adopting secondary or tertiary structures, thereby increasing the rigidity of the peptide. Moreover, because it lacks the surrounding residues present in the native protein, such a long and isolated peptide would be unlikely to fold correctly, reducing its ability to mimic the authentic epitope. For these reasons, two shorter peptides were selected, synthesized, and subsequently evaluated. Our results demonstrated high sensitivity and specificity for both Peptide 1 and Peptide 2 when tested with either urine or serum samples from patients indicated that synthesizing these molecules separately was indeed a judicious choice.

The recombinant K39 antigen (rK39), which is found within the Detect Rapid Test, is a protein consisting of multiple 39–amino acid repeats derived from a kinesin-like gene of *Leishmania infantum*. This kinesin-like gene is present in distinct *Leishmania* species and conserves the main B-cell epitopes among them. In this study, we used this kit as a commercial comparator antigen for ENDO. Although the kit showed good performance for the detection of leishmaniasis, its performance was lower when compared with the use of the recombinant protein. Certainly, further studies are required to confirm these preliminary findings. In addition, other parasite antigenic preparations, such as those derived from *L. infantum* species, could also be used as comparative antigens in our ELISA assays. In fact, several proteins are conserved among these parasite preparations, and many conserved B-cell epitopes are shared between them. In this context, the data can be considered satisfactory; however, additional experiments are necessary to further validate these results.

In addition to the importance of antigen selection for the diagnosis of leishmaniasis, the use of treatment-monitoring markers is also relevant, as they may serve as indicators of clinical cure and aid in patient follow-up. In this context, humoral follow-up after leishmaniasis treatment should be considered an important tool for monitoring therapeutic responses [81–83]. Although commercial kits show good sensitivity for detecting active disease, they are of limited effectiveness in distinguishing antibody levels before and after treatment, since moderate antibody reactivity is often observed in treated patients [84,85]. In our study, anti-rENDO antibody levels declined by approximately 50% within six months after patient treatment. This finding is noteworthy and suggests that this protein may be useful as a treatment-monitoring marker for TL. However, the sample size used in this longitudinal study was small and limited, and only a single post-treatment time point was evaluated. These limitations make it impossible to draw definitive conclusions regarding the effectiveness of ENDO as a biomarker for TL. Additionally, it was not possible to confirm the prognostic role of anti-rENDO antibodies in relation to patient cure, since comparative studies evaluating antibody dynamics in patients who did not achieve clinical improvement were not performed. Moreover, the absence of correlation with parasitological techniques, such as PCR, prevents validation of whether parasitological cure is associated with lower anti-ENDO antibody levels. In this context, the kinetics of the antibody response and potential fluctuations over time were not evaluated, and additional follow-up periods should be considered to better determine the usefulness of this recombinant protein in monitoring treatment response. Therefore, the results presented here should be interpreted with caution and considered preliminary, yet promising, regarding the potential use of this antigen for such biological purposes.

To summarize, rENDO and two derived synthetic peptides were tested in ELISA as diagnostic antigens for TL and reported high sensitivity and specificity for diagnosing TL. However, we concede that our study has several limitations that should be considered when interpreting the findings. First, it represents a preliminary, single-centre (Belo Horizonte) study with relatively small sample sizes in several subgroups, particularly malaria, HIV, and the treated ML follow-up cohort, which may limit the statistical power and generalizability of the results. Additionally, although a CRD panel was included, it comprised only a limited set of co-endemic infections, and therefore the full spectrum of potential cross-reactivity, especially with other protozoan and helminth infections, remains incompletely addressed. The use of stored samples, if details on storage duration and freeze–thaw history are incomplete, also introduces potential variability in antibody stability. Moreover, all patients had established CL or ML, and the performance of the antigens in early, asymptomatic, or subclinical infections was not assessed. The study also focused exclusively on linear B-cell peptides predicted *in silico*, without the more difficult evaluation of hypothetical conformational epitopes or alternative antigenic regions that may contribute to diagnostic performance *in vivo*. In addition, we did not compare the linear peptides in ELISA with the Detect Rapid Test (inBios), which detects antibodies against the conserved k39 antigen, whereas isolated linear peptides capture only a subset of epitopes. Thus, because peptide reactivity cannot be expected to correlate directly with responses to complex antigens used in commercial tests, we did not include a formal comparison between peptide ELISAs and the rapid test. Finally, although antibody decline after treatment can also suggest prognostic potential, follow-up was limited to a single six-month timepoint, preventing conclusions regarding long-term dynamics or relapse detection. Collectively, these limitations highlight the need for broader multicenter validation, larger and more diverse cohorts, and expanded antigenic testing to fully establish the biological utility of rENDO and its peptides against leishmaniasis. Regardless, our study data can be considered as providing preliminary proof-of-concept of the use of a urine-based ELISA – as an alternative to serum - using rENDO or their synthetic peptides as diagnostic candidates for TL.

## Supporting information

S1 Data 1) Amino acid sequence of ENDO protein>CAC9452939.1 endonuclease_III-putative [*Leishmania infantum*]. 2) BLAST for similarity of ENDO within *Leishmania.* 3) FASTA sequences of selected proteins from *Leishmania* species. 4) IEDB Emini surface accessibility prediction results for ENDO protein. 5) ABCpred results to predict B-cell epitope(s) of ENDO protein. 6) Clustal alignment of the amino acid sequences of ENDO proteins from different *Leishmania* species.
(PDF)
