# Supplementary material for: Comparison of a recombinant endonuclease III protein and its synthetic peptides in ELISA for the diagnosis of tegumentary leishmaniasis using human serum and urine samples: A preliminary study
Source: PLoS Negl Trop Dis. 2026 Jun 15;20(6):e0014440. doi: 10.1371/journal.pntd.0014440 (PMC13293512; doi:10.1371/journal.pntd.0014440)
Supplement: S1 Data — 1) Amino acid sequence of ENDO protein>CAC9452939.1 endonuclease_III-putative [Leishmania infantum]. 2) BLAST for similarity of ENDO within Leishmania. 3) FASTA sequences of selected proteins from Leishmania species. 4) IEDB Emini surface accessibility prediction results for ENDO protein. 5) ABCpred results to predict B-cell epitope(s) of ENDO protein. 6) Clustal alignment of the amino acid sequences of ENDO proteins from different Leishmania species. (PDF) [file pntd.0014440.s001.pdf]

Proteína 3

>CAC9452939.1

(ENDO)

>CAC9452939.1 endonuclease_III-__putative [Leishmania infantum]

MNKCSFTPPSNWAQLFARLEDYRKHLKAPVDTMGCHRLRDEYAPKEVQRFHTLVALMLSAQTKDVVTAAAMD
TLIKHGLTAQSVHAMTETELDKHICKVGFHNKKAKNIKEVAAILMKNYDGEVPREYAELIALPGVGPKMANL
FFQDADHRVIGIGVDTHVHRISQRYRWVPSTVKTPEDTRKALESWLPREHWGTINSLMVGLGQTVCTPLRPK
CGICELSDICPNAFKETQQKRLRAKAPLVEKEEPVSNKKRRK

Proteína 3 >CAC9452939.1
Blast, similarities with *Leishmania*

Description	Scientific Name	Max Score	Total Score	Query Cover	E value	Per. Ident	Acc. Len	Accession
putative endonuclease III [Leishmania infantum JPCM5]	Leishmania infantum JPCM5	541	541	100%	0.0	100.00%	258	XP_001463464.1
putative endonuclease III [Leishmania major strain Friedlin]	Leishmania major strain Friedlin	508	508	99%	0.0	94.55%	257	XP_001681168.1
putative endonuclease III [Leishmania mexicana MHOM/GT/2001/U1103]	Leishmania mexicana MHOM/GT/2001/U1103	501	501	100%	2e-178	93.41%	258	XP_003872687.1
hypothetical protein CUR178_07135 [Leishmania enriettii]	Leishmania enriettii	473	473	99%	3e-167	87.55%	261	KAG5484544.1
hypothetical protein GH5_07190 [Leishmania sp. Ghana 2012 LV757]	Leishmania sp. Ghana 2012 LV757	470	470	99%	4e-166	87.16%	261	KAG5510989.1
hypothetical protein JIQ42_06799 [Leishmania sp. Namibia]	Leishmania sp. Namibia	469	469	99%	1e-165	86.77%	261	KAG5506547.1
endonuclease III, putative [Leishmania panamensis]	Leishmania panamensis	468	468	99%	2e-165	86.38%	259	XP_010704245.1
hypothetical protein LSCM4_06696 [Leishmania orientalis]	Leishmania orientalis	468	468	99%	2e-165	86.77%	261	KAG5485990.1
endonuclease III, putative [Leishmania guyanensis]	Leishmania guyanensis	468	468	99%	2e-165	86.38%	259	CCM13298.1

putative endonuclease III [Leishmania braziliensis MHOM/BR/75/M2904]	Leishmania braziliensis MHOM/BR/75/M2904	466	466	99%	9e-165	85.99%	259	XP_001562623.1
endonuclease III, putative [Leishmania tarentolae]	Leishmania tarentolae	465	465	100%	3e-164	86.43%	259	GET86244.1
HhH-GPD superfamily base excision DNA repair protein [Leishmania braziliensis]	Leishmania braziliensis	464	464	99%	5e-164	85.60%	259	KAI5690634.1
hypothetical protein LSCM1_07526 [Leishmania martiniquensis]	Leishmania martiniquensis	462	462	99%	6e-163	85.27%	262	KAG5485442.1
hypothetical protein JKF63_06805 [Porcisia hertigi]	Porcisia hertigi	448	448	100%	2e-157	82.17%	259	KAG5510508.1
putative endonuclease III [Leptomonas pyrrhocoris]	Leptomonas pyrrhocoris	400	400	90%	2e-138	78.21%	272	XP_015655864.1
putative endonuclease III [Leptomonas seymouri]	Leptomonas seymouri	385	385	99%	2e-132	68.50%	273	KPI83344.1
HhH-GPD base excision DNA repair family protein [Leishmania donovani]	Leishmania donovani	347	347	63%	8e-119	100.00%	172	TPP42928.1

Proteína 3

>CAC9452939.1

Sequência FASTA das proteínas selecionadas BLAST- p - *Leishmania*

>XP_001463464.1 putative endonuclease III [Leishmania infantum JPCM5]

MNKCSFTPPSNWAQLFARLEDYRKHLKAPVDTMGCHRLRDEYAPKEVQRFHTLVALMLSAQTKDVVTAAAMDTLIKHGLTAQSVHAM
TETELDKHICKVGFHNKKAKNIKEVAAILMKNYDGEVPREYAELIALPGVGPKMANLFFQDADHRVIGIGVDTHVHRISQRYRWVPS
TVKTPEDTRKALESWLPREHWGTINSLMVGLGQTVCTPLRPKCGICELSDICPNAFKETQQKRLRAKAPLVEKEEPVSNKKRRK

>XP_001681168.1 putative endonuclease III [Leishmania major strain Friedlin]

MNKRSFTPPSNWAQLFARLEDYRKHLKAPVDTMGCHRLRDETAPKEVQRFHTLVALMLSAQTKDVVTAAAMDTLIKRELTVQSVHAM
TETELDKHICKVGFHNTKARNIKEVAAILMKNYDGKVPREYAELIALPGVGPKMANLFFQDADHRVIGIGVDTHVHRISQRYRWVPS
TVKTPEDTRKALESWLPREHWGTINSLMVGLGQTVCTPLRPKCDICELSDICPNAFKERRQKRLRAKAPLMEKEEPVSHRKRR

>XP_003872687.1 putative endonuclease III [Leishmania mexicana MHOM/GT/2001/U1103]

MNKRSFTPPSNWGQLFARLEDYRKHLKAPVDTMGCHRLRDENAPKEVQRFHTLVALMLSAQTKDVVTAAAMDALIKRGLTAQSVHAM
TERELDKHICKVGFHNTKARNIKEVAAILMKDYDGKVPREYAEVIALPGVGPKMANLFFQDADHRVIGIGVDTHVHRISQRYRWVPS
TVKTPEDTRKALESWLPLEHWGTINSLMVGLGQTVCTPLRPKCDICELSGICPNAFKETQQKRLRAKGLLVEKEEPVSHKKRRK

>XP_001562623.1 putative endonuclease III [Leishmania braziliensis MHOM/BR/75/M2904]

MSKHSFTPPSNWAQLFARLEDYRKHLLAPVDTMGCHRLHDENAPKEVQRFQTLVALMLSAQTKDIVTATAMDALIKRGLTAQSIHAM
TTTELDMHICKVGFHNTKVKHIKEVAAILIKDYGGKVPREYEELIALPGVGPKMANLFFQDADHRTVGIGVDTHVHRISQRYRWVPS
TVKTPEDTRKALESWLPQKHWGTINSLMVGLGQTVCTPLYPKCGICELSDICPNAFKEVQQKGLRTKAPTERRQEPVPQKKRRIK

>TPP42928.1 HhH-GPD base excision DNA repair family protein [Leishmania donovani]

MSFTPPSNWAQLFARLEDYRKHLKAPVDTMGCHRLRDEYAPKEVQRFHTLVALMLSAQTKDVVTAAAMDTLIKHGLTAQSVHAMTET
ELDKHICKVGFHNKKAKNIKEVAAILMKNYDGEVPREYAELIALPGVGPKMANLFFQDADHRVIGIGVDTHVHRISQRYGGYQAP

Proteína 3

>CAC9452939.1

PREDIÇÃO IEDB: Center position: 7

Window size:14 Threshold:1.000

IEDB

MNKCSFTPPSNWAQLFARLEDYRKHLKAPVDTMGCHRLRDEYAPKEVQRFHTLVALMLSAQTKDVV
TAAAMDTLIKHGLTAQSVHAMTETELDKHICKVGFHNKKAKNIKEVAAILMKNYDGEVPREYAELI
ALPGVGPKMANLFFQDADHRVIGIGVDTHVHRISQRYRWVPSTVKTPEDTRKALESWLPREHWGTI
NSLMVGLGQTVCTPLRPKCGICELSDICPNAFKETQQKRLRAKAPLVEKEEPVSNKKRRK

Proteína 3 >CAC9452939.1

ABCpred Threshold 0.85, window size: 16

Tabular result: predicted B-cell epitope. The predicted B cell epitopes are ranked according to their score obtained by trained recurrent neural network. Higher score of the peptide means the higher probability tobe as epitope. Al the peptides shown here are above the threshold value chosen.

Rank	Sequence	Start position	Score
1	NKCSFTPPSNWAQLFARL	2	0.88
2	HLKAPVDTMGCHRLRDEY	25	0.87
3	PKEVQRFHTLVALMLSAQ	44	0.86
4	IGIGVDTHVHRISQRYRW	154	0.85

Proteína 3

>CAC9452939.1

CLUSTAL OMEGA COM TODOS OS DADOS DE PREDIÇÃO – *Leishmania*

```
Leishmania infantum (CAC9452939.1)      MNKCSFTPPSNWAQLFARLEDYRKHLKAPVDTMGCHRLRDEYAPKEVQRFHTLVALMLSAQTKDVVTAAAMDTLIKHGLTAQSVHAMTETELDKHICKVGFHNKKAKNIKEVAAILMKNY     120
Leishmania infantum (XP_001463464.1)    MNKCSFTPPSNWAQLFARLEDYRKHLKAPVDTMGCHRLRDEYAPKEVQRFHTLVALMLSAQTKDVVTAAAMDTLIKHGLTAQSVHAMTETELDKHICKVGFHNKKAKNIKEVAAILMKNY     120
Leishmania donovani (TPP42928.1)        ---MSFTPPSNWAQLFARLEDYRKHLKAPVDTMGCHRLRDEYAPKEVQRFHTLVALMLSAQTKDVVTAAAMDTLIKHGLTAQSVHAMTETELDKHICKVGFHNKKAKNIKEVAAILMKNY     117
Leishmania major (XP_001681168.1)       MNKRSFTPPSNWAQLFARLEDYRKHLKAPVDTMGCHRLRDETAPKEVQRFHTLVALMLSAQTKDVVTAAAMDTLIKRELTVQSVHAMTETELDKHICKVGFHNTKARNIKEVAAILMKNY     120
Leishmania mexicana (XP_003872687.1)    MNKRSFTPPSNWGQLFARLEDYRKHLKAPVDTMGCHRLRDENAPKEVQRFHTLVALMLSAQTKDVVTAAAMDALIKRGLTAQSVHAMTERELDKHICKVGFHNTKARNIKEVAAILMKDY     120
Leishmania braziliensis (XP_001562623.1) MSKHSFTPPSNWAQLFARLEDYRKHLLAPVDTMGCHRLHDENAPKEVQRFQTLVALMLSAQTKDIVTATAMDALIKRGLTAQSIHAMTTTELDMHICKVGFHNTKVKHIKEVAAILIKDY     120
                                        ********.************* ***********:** ********:*************:***:***:***: **.**:****  *** *********.*.::********:*:*

Leishmania infantum (CAC9452939.1)      DGEVPREYAELIALPGVGPKMANLFFQDADHRVIGIGVDTHVHRISQRYRWVPSTVKTPEDTRKALESWLPREHWGTINSLMVGLGQTVCTPLRPKCGICELSDICPNAFKETQQKRLRA     240
Leishmania infantum (XP_001463464.1)    DGEVPREYAELIALPGVGPKMANLFFQDADHRVIGIGVDTHVHRISQRYRWVPSTVKTPEDTRKALESWLPREHWGTINSLMVGLGQTVCTPLRPKCGICELSDICPNAFKETQQKRLRA     240
Leishmania donovani (TPP42928.1)        DGEVPREYAELIALPGVGPKMANLFFQDADHRVIGIGVDTHVHRISQRYGGYQAP-----------------------------------------------------------------     172
Leishmania major (XP_001681168.1)       DGKVPREYAELIALPGVGPKMANLFFQDADHRVIGIGVDTHVHRISQRYRWVPSTVKTPEDTRKALESWLPREHWGTINSLMVGLGQTVCTPLRPKCDICELSDICPNAFKERRQKRLRA     240
Leishmania mexicana (XP_003872687.1)    DGKVPREYAEVIALPGVGPKMANLFFQDADHRVIGIGVDTHVHRISQRYRWVPSTVKTPEDTRKALESWLPLEHWGTINSLMVGLGQTVCTPLRPKCDICELSGICPNAFKETQQKRLRA     240
Leishmania braziliensis (XP_001562623.1) GGKVPREYEELIALPGVGPKMANLFFQDADHRTVGIGVDTHVHRISQRYRWVPSTVKTPEDTRKALESWLPQKHWGTINSLMVGLGQTVCTPLYPKCGICELSDICPNAFKEVQQKGLRT     240
                                        .*:***** *:*********************.:***************    :

Leishmania infantum (CAC9452939.1)      KAPLVEKEEPVSNKKRRK-       258          Similarity
Leishmania infantum (XP_001463464.1)    KAPLVEKEEPVSNKKRRK-       258             100%
Leishmania donovani (TPP42928.1)        -------------------       172             100%
Leishmania major (XP_001681168.1)       KAPLMEKEEPVSHRKRR--       257            94,55%
Leishmania mexicana (XP_003872687.1)    KGLLVEKEEPVSHKKRRK-       258            93,41%
Leishmania braziliensis (XP_001562623.1) KAPTERRQEPVPQKKRRIK       259            85,99%
```
