# Supplementary material for: Comparison of a recombinant endonuclease III protein and its synthetic peptides in ELISA for the diagnosis of tegumentary leishmaniasis using human serum and urine samples: A preliminary study
Source: PLoS Negl Trop Dis. 2026 Jun 15;20(6):e0014440. doi: 10.1371/journal.pntd.0014440 (PMC13293512; doi:10.1371/journal.pntd.0014440)
Supplement: Attachment — Submitted filename: 2026 02 25 PNTD-D-26-00207 reviewers attachment.pdf [file pntd.0014440.s002.pdf]

I. Summary of the research

Authors use leishmania cytoplasmic protein- endonuclease III (ENDO), to develop a set of recombinant protein (rENDO) and pair of peptide antigens specific ELISA for diagnostic and monitoring using patient serum and urine samples in the context of tegumentary leishmaniasis (consisting of cutaneous leishmaniasis and mucosal leishmaniasis). Authors discuss the limitations of current gold standard for diagnosis dependent of parasitological test and establish the need for an immunological technique oriented and cost-effective diagnostic assay. Assay shows strong sensitivity and specificity metric for the recombinant protein and peptide antigen and outperforms soluble leishmania antigen (SLA) obtained from *L. braziliensis* strain. The assay also demonstrates chosen antigen's ability to selectively diagnose CL and ML over other cross-reactive diseases- malaria, chagas, HIV, and leprosy.

II. Major issues:

1. Cross reactivity data

    a. Was *L. brazeiliensis* the only species causing TL included in the study cohort? Were other TL causing species endemic to the area identified, recorded using PCR and included in the study? If so, the paper could benefit sharing the list of leishmania species identified to understand species dependent assay performance.

    b. The data lacks in mentioning if the ELISA was able to distinguish between CL and ML in an unknown sample. Since CL and ML can be caused by different species of leishmania- is strain identification using this assay ideal to establish clinical treatment protocol post diagnosis?

    c. Method section is missing the technique used to identify malaria cases in the cross-reactive diseases (CRD) group. Figure legend often misses to mention if the malaria cases were included in the analysis of the CRD group.

    d. The method section mentions that Detect™ Rapid Test for Visceral Leishmaniasis was used (Later it is mentioned it was used to establish the data in table 3) but misses to mention if this assay was used for inclusion criteria- to confirm TL or exclude VL in the study cohort.

2. Additionally, it is unclear from table 3 if the HC and CRD samples were used to assess false-positive and true-negative.

3. Graph in Fig 5A detecting CL cases in serum is significantly below the cutoff, the figure legend attributes this to peptide 2 but results discussion attributes this data to SLA. Please clarify.
4. Please include the graphical data showing variability of the 15 ML patient samples used for antibody follow-up in treated patients' portion of the study. The results describes the average IgG reduction in the samples but is missing the variability data.
5. It is difficult to confidently claim anti-rENDO antibodies can be used as a prognostic biomarker since the data does not include information on how the biomarker behaves in cases where patient doesn't show clinical improvement or if the patient takes a different disease course. There is also lack of data showing correlation of these antibodies with a standard clinical evaluation e.g. PCR values detecting parasitic load vs anti-rENDO antibodies.

III. Minor issues

1. B-cell epitope selection:
    a. B cell peptide chosen for the assay are from endonuclease III (ENDO) which is a cytoplasmic protein for leishmania parasite. It will be beneficial to include a short discussion of why a cytoplasmic protein instead of surface antigen was chosen for this ELISA assay and potential mechanism on how this protein may be presented to the host immune system for B-cell antibody development.
    b. The paper could benefit from discussion of how conserve these linear B-cell epitopes are across multiple leishmania species as shown in the supplementary data and its implication in clinical treatment decision making post diagnosis.
2. SLA using for this study was isolated only from *L. Brasiliensis*, the paper lacks discussion of rationale for choosing only this ML causing species instead of multiple parallel TL causing species. Would the authors expect the assay performance to change based on SLA from other species?
3. The study could benefit from a sample stability testing- especially for urine samples due to their acidic nature, to see how long the antigen binding antibodies stays sable in the sample before analysis.
