# Supplementary material for: Comparison of a recombinant endonuclease III protein and its synthetic peptides in ELISA for the diagnosis of tegumentary leishmaniasis using human serum and urine samples: A preliminary study
Source: PLoS Negl Trop Dis. 2026 Jun 15;20(6):e0014440. doi: 10.1371/journal.pntd.0014440 (PMC13293512; doi:10.1371/journal.pntd.0014440)
Supplement: Attachment — Submitted filename: Detailed List of responses to the Reviewers.doc [file pntd.0014440.s005.doc]

**List of Responses to the Editor and Reviewers**

**METHODS**

**Reviewer #1**

The study followed a single-center, case-control, phase I diagnostic accuracy design using archived samples. However, this is not explicitly stated in the methods.
**Answer**: Firstly, we would like to thank the Reviewer for the evaluation of our article. In the revised version of the manuscript, we have worked thoroughly to answer all queries raised, to improve the quality of our study, as well as make it suitable to be published in PloS Neglected Tropical Diseases. According to the Reviewer´ suggestion, we have included this information in the revised text (lines 160-163 and 408-410), aiming to make clearer the information for the readers.

2. The manuscript does not include a participant flow diagram as recommended by STARD. Missing STARD elements include Flow diagram of patient inclusion; Description of missing or excluded samples; Justification of sample size; Description of blinding procedures; Handling of indeterminate results.
**Answer**: We must clarify the reviewer with new information added to the distinct sections of the study, mainly emphasizing the limitations of the experimental procedures and conclusions enrolled. Since we have used a well-controlled urine and serum panel, which was pre-existing and archival in our laboratory, no recruitment of patients was performed. All selected samples from our panel were tested individually and such information included in the Methodology section, see lines 160-172. Thus, we do not believe that specific STARD elements are required for this study.

3. Please clarify whether the ELISA interpretation was performed blindly.
**Answer**: The information was included in the revised manuscript. Please see lines 242-247: “All urine and serum samples were codified previously before used, being a blind study conducted by our group. Samples were evaluated using the same reagents (lots, dilutions, etc) to reduce possible experimental variations. Two different ELISA procedures were done by independent people using the same serum and urine samples, and results obtained were similar between them (data not shown). Finally, a Bland-Altman plot analysis showed no significant difference in OD values between the duplicates performed”.

4. Although TL diagnosis was based on clinical evaluation combined with smear microscopy and PCR, the manuscript does not clearly state: Whether all cases were PCR-confirmed; Whether discrepant results occurred;

**Answer**: we thank the reviewer for the clarification request. We have now included the information in the revised text. Please see lines 173-189: as follows - “TL cases, here represented by CL and ML patients, were diagnosed by clinical evaluation by clinicians with expertise and laboratory assays of skin and/or tissue biopsies. Giemsa-stained smears from lesion fragments and Polymerase Chain Reaction (PCR) to detect *L. braziliensis* kDNA were used as diagnostic tools [25, 26]. Briefly, the parasite kDNA was extracted from samples through the QIAamp^®^ DNA Blood Mini Kit (Qiagen^®^, MD, USA). It was used to amplify a specific *L. braziliensis* kDNA region (150 base pairs). The following primers were used in the reactions: 5´-GGGKAGGGGCGTTCTSCGAA-3´ (*Forward*) and 5´-SSSWCTATWTTACACCAACCCC-3´ (*Reverse*). *Leishmania* kDNA was also extracted of a reference *L. braziliensis* strain (MHOM/BR/2002/LPC-RPV) used as positive control; while sterile ultrapure water was used as negative control. The following reagents were employed in the reactions: 2 mM MgCl_2_, 200 µM dNTPs, 0.6 µM of each primer (Sigma-Aldrich, USA), 1 UI Taq DNA polymerase with specific buffer (Invitrogen, USA), and 20 ng DNA templates. The program applied was: (step 1) 94ºC for 10 min; (step 2) 60ºC for 30 seconds; (step 3) 72ºC for 30 seconds; (step 4) 94ºC for 30 seconds and go to step 2 for 42 times; (step 5) 72ºC for 10 min (Pereira et al., 2014). All CL and ML patients presented positive PCR result. None of them had any previous history of other infectious disease and did not receive any treatment at the time of urine and serum collection”.

**Reviewer #2**

Major Issues:
1. Cross reactivity data
a. Was L. braziliensis the only species causing TL included in the study cohort? Were other TL causing species endemic to the area identified, recorded using PCR and included in the study? If so, the paper could benefit sharing the list of leishmania species identified to understand species dependent assay performance.
**Answer**: We also would like to thank the Reviewer for the evaluation of our manuscript. In the revised version of the article, we have worked thoroughly to answer all queries raised, aiming to improve the quality of our work and make it more suitable for publication. Regarding the question raised, the unique *Leishmania* species detected in the tegumentary leishmaniasis patients enrolled in the study was *Leishmania braziliensis*. We have used molecular techniques to prove such information, and this is included in the text (lines 176-189).

b. The data lacks in mentioning if the ELISA was able to distinguish between CL and ML in an unknown sample. Since CL and ML can be caused by different species of leishmania- is strain identification using this assay ideal to establish clinical treatment protocol post diagnosis?
**Answer**: We understand the criticism of the Reviewer. Consequently, we have included a new sentence about this theme in the Discussion section (please see lines 389-410): as follows - “In general, CL and ML exhibit distinct clinical manifestations in the patients, differing in location, progression, and severity. CL is typically confined to the skin, presenting as one or more painless ulcers with indurated borders at exposed sites, and it often follows a chronic but self-limited course with spontaneous healing and scar formation. In contrast, ML involves the mucous membranes of the upper respiratory tract, particularly the nasal and oropharyngeal regions, and usually develops months to years after an initial cutaneous lesion due to parasite dissemination. Unlike the localized and relatively benign nature of cutaneous disease, mucosal involvement is progressive and destructive, characterized by chronic inflammation, ulceration, and significant tissue loss, leading to symptoms such as nasal obstruction, epistaxis, dysphonia, and dysphagia. Patients with CL tend to have a higher parasite load with moderate immune response; mucosal leishmaniasis is associated with low parasite burden but an exaggerated inflammatory response, contributing to severe tissue damage and the need for prompt systemic treatment. Such facts contribute to that the clinicians have conditions to differentiate between CL and ML in patients. In this context, data presented here can be considered satisfactory, since the ENDO antigen was able to identify both disease cases, when humoral reactivity was evaluated. Such fact can add information for the safe and precise diagnosis of leishmaniasis, in contrast to other infectious diseases in which symptomatology is also similar to TL”.

c. Method section is missing the technique used to identify malaria cases in the cross-reactive diseases (CRD) group. Figure legend often misses to mention if the malaria cases were included in the analysis of the CRD group.
**Answer**: We apologize for this mistake. The information was included in the text (lines 192-193). Legends now also contain information regarding malaria subjects.

d. The method section mentions that Detect™ Rapid Test for Visceral Leishmaniasis was used (Later it is mentioned it was used to establish the data in table 3) but misses to mention if this assay was used for inclusion criteria- to confirm TL or exclude VL in the study cohort.
Answer: We apologize for this mistake. We have tried to improve the description of the use of kit in the study. Please see lines 249-254: as follows - “Comparative analysis using a commercial kit. The Detect*™* Rapid Test (InBios International Inc., USA), which is an immunochromatographic test for the qualitative detection of antibodies against the conserved k39 protein from *Leishmania* spp., was used according to the manufacturer’s instruction. The kit was used as an antigen control against urine and serum samples of the TL patients to evaluate the performance of the rENDO protein”. In other sentences the content was also altered, and modifications were marked in the revised text.

2. Additionally, it is unclear from table 3 if the HC and CRD samples were used to assess false-positive and true-negative.
**Answer**: We have corrected the information in Table 3. Indeed, only samples from TL patients were used for comparison of the rENDO-ELISA and the commercial kit, as stated in the table legend and in the Materials and Methods section. For this reason, the kappa coefficient of agreement between tests could not be calculated, since TN and FP are necessarily dropped to “0” results, resulting in all cases kappa=0.

3. Graph in Fig 5A detecting CL cases in serum is significantly below the cutoff, the figure legend attributes this to peptide 2 but results discussion attributes this data to SLA. Please clarify.
**Answer**: We apologize for this mistake in the indication of the figures. In fact, Figures 2, 3, 4 and 5 refer to results obtained using ENDO protein, Peptide 1, Peptide 2 and SLA as antigens, respectively. The mistake has been corrected throughout the revised Results and Discussion.

4. Please include the graphical data showing variability of the 15 ML patient samples used for antibody follow-up in treated patients’ portion of the study. The results describe the average IgG reduction in the samples but is missing the variability data.
**Answer**: We thank the reviewer for detecting this omission, and therefore have now included a new Figure 6 to show the variability, with a new Figure legend added to explain the graph. See Figure 6 and lines 653-657.

5. It is difficult to confidently claim anti-rENDO antibodies can be used as a prognostic biomarker since the data does not include information on how the biomarker behaves in cases where patient doesn’t show clinical improvement or if the patient takes a different disease course. There is also lack of data showing correlation of these antibodies with a standard clinical evaluation e.g. PCR values detecting parasitic load vs anti-rENDO antibodies.
**Answer**: We understand the criticism of the Reviewer in highlighting this limitation, which we concede and discuss. As consequence, a sentence was included in the Discussion about this topic. Please see lines 551-560: as follows - “Additionally, it is not possible to confirm the prognostic role of anti-rENDO antibodies with the patient cure; since a comparative study evaluating if these antibody levels could reduce or not in other patients in which any clinical improvement was reached, as well as by absence of correlation with results of parasitological techniques, such as PCR, to validate if the parasitological cure will be related with lower levels of anti-ENDO antibodies”.

Minor issues
1. B-cell epitope selection:
a. B cell peptide chosen for the assay are from endonuclease III (ENDO) which is a cytoplasmic protein for leishmania parasite. It will be beneficial to include a short discussion of why a cytoplasmic protein instead of surface antigen was chosen for this ELISA assay and potential mechanism on how this protein may be presented to the host immune system for B-cell antibody development.
**Answer**: In concordance, we have included a sentence about this topic in the revised manuscript (lines 482-494): as follows – ‘’ Recombinant Leishmania antigens have been extensively evaluated as diagnostic tools for leishmaniasis, and cytoplasmic proteins have also been explored for this purpose. Antigens such as the A2 protein [72, 73], enolase [74], and heat shock proteins [75, 76], among others, have been cloned, and their recombinant forms have been used in ELISA and other diagnostic platforms to detect leishmaniasis. These antigens are primarily metabolic and structural proteins associated with parasite survival and virulence, as well as stress-related proteins and molecular chaperones. Notably, the presence of cytoplasmic proteins in the amastigote stage allows closer interaction with the host immune system, particularly due to the reduced abundance of membrane-associated antigens in this life stage of Leishmania [77]. ENDO, another cytoplasmic parasite protein, has previously been recognized by antibodies in patients with active visceral leishmaniasis (VL) [23], and in the present study, its recombinant form was also shown to exhibit antigenicity suitable for the identification of leishmaniasis cases.’’

b. The paper could benefit from discussion of how conserve these linear B-cell epitopes are across multiple leishmania species as shown in the supplementary data and its implication in clinical treatment decision making post diagnosis.
**Answer**: In concordance, we have included a sentence about this theme. Please see lines 441-450: as follows - ‘’Accurate diagnosis of leishmaniasis is essential towards efficient control of this NTD, but it presents problems related to the sensitivity and/or specificity of the tests [52]. In fact, antibody cross-reactions can occur mainly with other Trypanosomatides antigens, and the use of more refined antigens, such as synthetic peptides, could limit this cross-reactivity [53]. Additionally, synthetic peptides are easier to produce and standardize. The data from our present study are encouraging, since the use of synthetic peptides shows good sensitivity and specificity like the use of SLA. This finding can be explained by the fact that amino acid sequences able to cause cross-reactivity with other antibodies are eliminated and thus it may be possible to select and construct only peptides specific to detect TL or VL, or both diseases [54].’’

2. SLA using for this study was isolated only from L. braziliensis, the paper lacks discussion of rationale for choosing only this ML causing species instead of multiple parallel TL causing species. Would the authors expect the assay performance to change based on SLA from other species?
**Answer**: To clarify, most of the Leishmania antigens are conserved between parasite species causing tegumentary or visceral leishmaniasis. In house studies using distinct parasite antigenic preparations derived from species such as Leishmania infantum, Leishmania braziliensis, Leishmania major, and Leishmania amazonensis show a 70-80% similarity in SDS-PAGE between the main antigens, and when such preparations are used in ELISA as control antigens, optical density values obtained using canine or human sera from leishmaniasis are too similar, demonstrating that there is no relevance difference between them to prepare such antigenic extracts and their use in ELISA. In addition, we have highlighted in the Introduction that Leishmania braziliensis is the most prevalent parasite species causing tegumentary (cutaneous and mucosal) leishmaniasis at Brazil, see lines 95-101. This fact highlights the antigenic preparation using this parasite species and its use in comparative ELISA in our study.

3. The study could benefit from a sample stability testing- especially for urine samples due to their acidic nature, to see how long the antigen binding antibodies stays sable in the sample before analysis.

**Answer**: We thank the reviewer for this question on the lability of urine samples. In fact, since our group has worked a lot with urine as a biological sample, we have performed stability studies regarding the storage of this biological fluid in temperatures such as 4^o^C. In works published in the literature (doi: 10.1016/j.actatropica.2024.107326 and doi: 10.3390/v15091877) similar stability studies were done and a period of six months indicated maintenance of specific humoral response in the tested samples, when the diagnosis of tegumentary leishmaniasis and COVID-19, respectively, were evaluated. We have included a sentence about this topic in the Discussion (lines 457-464): as follows – ‘’The advantages of urine as an analyte include no invasive collection by the patient and urinary antibodies have been shown to be stable for a long period of time. Recently, Câmara et al. [34] performed a stability test using 18 urine samples from TL patients, and no significant variation in the ELISA optical density values was found in relation to of the levels of anti-LiHyV antibodies, when samples were stores for six months at 4oC. Similar findings have been also showed by others regarding the stability of urinary antibodies generated during different diseases [49, 62, 63]. In our study, a pilot stability assay showed also that anti-ENDO antibodies remained at similar levels even after three months storage at 4oC. Thus, the promising stability data reflect the possible long-term use of urine as a biological analyte for the immunodiagnosis of leishmaniasis.’’

**RESULTS**

**Reviewer #1**

1. The study reports:
100% sensitivity and specificity for rENDO (serum and urine)
100% sensitivity and specificity for Peptide 2
AUC = 1.0 in multiple ROC analyses
While promising, this perfect diagnostic separation is uncommon in tissue leishmaniasis studies, particularly for cutaneous leishmaniasis, where antibody titers are often low.
Results should be interpreted with caution and conclusions should be considered with moderation.
**Answer**: In concordance, in several sentences of the study we have clarified this point. As example, a sentence was included in the Introduction, lines 133-139: as follows - “Our main goal was to develop a potential new antigenic candidate as a diagnostic and/or prognostic marker for TL for future studies. The data presented herein are preliminary and the serum and urine panels used in the experiments were pre-existing archival samples, well-controlled and known. Such factors may mask the true diagnostic accuracy obtained, and when new samples are tested, whether derived from different locations or countries or collected in the field, they may result in reduced sensitivity and specificity values, as will be reported in our work.’’

2. The follow-up cohort (n = 15 patients with mucosal leishmaniasis, LM) showed a reduction in antibody levels six months after treatment. However, some important limitations should be considered. First, the methodology does not clearly describe how this longitudinal comparison was performed. Second, it is unclear whether the 15 LM samples correspond to a subset of the original 30 LM cases included in the diagnostic analysis or whether they represent a distinct group. If only half of the original LM cohort was included, the rationale for excluding the remaining samples should be explicitly stated. Third, the sample size is small, which substantially limits the strength of any conclusions about post-treatment dynamics. Furthermore, only a single post-treatment time point (six months) was evaluated, preventing assessment of the kinetics of antibody reduction or possible fluctuations over time.
**Answer**: We apologize for these errors, which have now been corrected in the revised manuscript throughout the methods and explanatory result, see lines 160 -195, 256-263, and Results throughout.

No protein and synthetic peptides stability or assay stability studies were reported. Therefore, it cannot be ruled out that the observed reduction in antibody detection may be partially attributed to decreased antigen stability or assay performance, rather than a true biological reduction in antibody levels. This concern is particularly relevant because the SLA-based assay continued to demonstrate persistent reactivity, suggesting that antibody responses may not decrease uniformly across antigens. Finally, there is insufficient evidence to support the authors' hypothesis that these assays can serve as markers for treatment monitoring.
**Answer**: We have also included new sentences in the Discussion section about this theme, mainly with relation to the limitations of the work., see lines 441-450, 508-526 (peptides) and563-588 (limitations). We would dispute the issue of peptide and recombinant protein stability; as long as both are produced under good manufacturing conditions that eliminates the possibility of microbial contamination and the preparations contain preservatives and are stored under frozen conditions, there should be no issues concerning stability. Indeed, such issues do not arise even for peptides/recombinant proteins used in licensed human (non-Leish.) vaccines

3. The manuscript compares rENDO performance with the Kalazar Detect™ Rapid Test, which is designed for visceral leishmaniasis (VL), not TL. This comparison may not be methodologically appropriate and should be interpreted cautiously. If retained, it should be described as exploratory.
**Answer**: We understand the point-of-view of the Reviewer and agree that it is wholly exploratory given the limitations of the study with archival samples. However, since the kit is derived from a conserved *Leishmania* protein present in cutaneous and viscerotropic species, k39, we infer that it could be also used as an additional comparative antigen in our work, mainly due to the absence of a similar kit applied to diagnose tegumentary leishmaniasis. Such information was included in the Discussion, see lines 527-538. In several sentences of the Discussion, the limitations of the experiments were included, and conclusions were modified to an exploratory context to search for new antigens.

**Reviewer #2**

The paper is missing a few clarifications from the methods describing the testing of disease in patient cohorts. Additionally, the paper is missing the graphical data for antibody follow-up following treatment portion of the study.

**Answer**: We have made these modifications as requested, see answers to comments above.

**CONCLUSIONS**

**Reviewer #1**

The study presents promising preliminary data suggesting that rENDO and derived synthetic peptides may have high diagnostic accuracy for tegumentary leishmaniasis using both serum and urine samples. However, the extraordinary diagnostic performance reported must be interpreted with caution due to the case–control design, limited sample size, and absence of validation cohorts.
**Answer**: We are in concordance with the Reviewer´ comments, and we have modified the text and proposed this as a preliminary study with a small cohort of archival samples. This is reflected in the title of the paper, within the abstract and throughout the discussion.

With improved methodological transparency, strengthened discussion of limitations, moderated conclusions, and closer adherence to STARD guidelines, the manuscript could make a valuable contribution to the field of TL diagnostics.

**Answer**: We believe that we have now clarified the methodology, moderated our conclusions significantly and provided extensive limitations to the study; as regards the STARD guidelines, we believe these are not wholly relevant as discussed above. We would like to extend this pilot study to comply with STARD guidelines, but this is a future and expanded clinical study.

**Reviewer #2**

It is difficult to confidently claim anti-rENDO antibodies can be used as a prognostic biomarker since the data does not include information on how the biomarker behaves in cases where patient doesn’t show clinical improvement or if the patient takes a different disease course. There is also lack of data showing correlation of these antibodies with a standard clinical evaluation e.g. PCR values detecting parasitic load vs anti-rENDO antibodies.

**Answer**: In concordance, we have remodeled the discussion about a possible prognostic role of rENDO, highlighting the limitations of the study, mainly regarding the methodological procedures and ELISA performed. See Discussion , lines 539-560, and elsewhere throughout the discussion.

**Summary and General Comments**

**Reviewer #1**

The manuscript presents a relevant and innovative diagnostic approach for tegumentary leishmaniasis (TL), evaluating a recombinant endonuclease III protein (rENDO) and two synthetic peptides in ELISA platforms using both serum and urine samples. The inclusion of urine as a non-invasive biological sample is a notable strength and adds translational value. However, the extraordinarily high diagnostic performance reported (100% sensitivity and specificity, AUC = 1.0 in multiple analyses) requires careful methodological clarification and a more cautious interpretation. The study has scientific merit but requires improvements in methodological transparency, statistical robustness, and alignment with diagnostic accuracy reporting standards.
Strengths: Innovative non-invasive urine-based ELISA. Rational antigen selection based on immunoproteomics. Inclusion of cross-reactive disease panel. Evaluation of synthetic peptides. Preliminary longitudinal assessment.
**Answer**: We thank the careful evaluation of our article. We believe that we have now modified the manuscript significantly to address concerns and to make the article clearer, and mainly highlighting the limitations of the work, such as the use of a well-controlled and known panel of urine and serum samples existing in our laboratory. All limitations were included in the revised text.

Limitations:
Incomplete adherence to STARD reporting standards. The manuscript does not fully comply with all items recommended by the STARD checklist for reporting diagnostic accuracy studies, particularly regarding participant flow, blinding procedures, and detailed methodological transparency.
**Answer**: We have included new information in the sections of the study. Since we have used a well-controlled urine and serum panel, which was pre-existing in our laboratory, no recruitment of patients was performed. All selected samples from our panel were tested individually by distinct professionals. Such information was included in the Methodology.

Single-centre design. The study was conducted at a single centre, which may limit the generalizability of the findings to other epidemiological settings or patient populations.
Small subgroup sample sizes. Although the overall sample size is reasonable for a phase I exploratory study, several subgroups are relatively small. This may reduce statistical robustness and increase uncertainty in subgroup-specific performance estimates.
**Answer**: We have included this information and limitations of the work (see answers above). Certainly, additional studies using rENDO as antigen against samples from other laboratories and collected in the field will be developed to confirm the real diagnostic potential of this recombinant antigen. The present study is only the initial mention of the possibility of testing eENDO (and peptides) in future works.

Insufficient methodological detail. The study design, participant selection process, and reference standards for each diagnostic group are not described in sufficient detail. Greater transparency is required regarding inclusion criteria, diagnostic confirmation methods, and whether test interpretation was performed blinded to clinical classification.
**Answer**: We have tried to improve the quality of presentation of the information in several sections of the work, modifying all them according to the Reviewers´ suggestions, as discussed above in responses to questions raised. We believe these changes have markedly improved the manuscript over the initial submission.

Perfect AUC values with potential risk of overestimation. The reporting of AUC values equal to 1.0 raises concern about potential overestimation of diagnostic performance.

**Answer**: In fact, since we used well-controlled and known samples to verify the potential of the antigens for leishmaniasis, we believe that the data are robust. Certainly, when the antigens, such as rENDO, were tested against urine and serum samples collected in other regions or in field, the diagnostic accuracy will be reduced. However, preliminary data are encouraging regarding the possibility of this antigen being applied as diagnostic candidate in future laboratorial trials.

**Reviewer #2**

Authors use Leishmania cytoplasmic protein- endonuclease III (ENDO), to develop a set of recombinant protein (rENDO) and pair of peptide antigens specific ELISA for diagnostic and monitoring using patient serum and urine samples in the context of tegumentary leishmaniasis (consisting of cutaneous leishmaniasis and mucosal leishmaniasis). Authors discuss the limitations of current gold standard for diagnosis dependent of parasitological test and establish the need for an immunological technique oriented and cost-effective diagnostic assay. Assay shows strong sensitivity and specificity metric for the recombinant protein and peptide antigen and outperforms soluble Leishmania antigen (SLA) obtained from L. braziliensis strain. The assay also demonstrates chosen antigen's ability to selectively diagnose CL and ML over other cross-reactive diseases- malaria, chagas, HIV, and leprosy. However, the diagnostic test lacks the ability to distinguish between Leishmania species that can affect clinical treatment protocol that is usually followed after a positive diagnostic test. Additionally, the evidence shown in the paper do not support the claim of the potential use of anti-rENDO antibodies as a prognostic biomarker for TL.

**Answer**: We thank the rigorous evaluation of the Reviewer. In fact, data are preliminary and such facts are well-described in this revised manuscript describing the study. In addition, we have modified the sections discussing the prognostic role of rENDO by including the limitations pointed out by both Reviewers. Regarding the parasite species, we have also included the fact that *Leishmania braziliensis* is responsible by more than 90% cases of tegumentary leishmaniasis at Brazil, which was the focus of our study and where urine and serum samples were collected. Certainly, the evaluation of rENDO against samples from tegumentary leishmaniasis patients in which other *Leishmania* species were responsible by disease, which not *L. braziliensis*, could be developed mainly in countries where such parasite species are found.

We thank both reviewers and PLOS NTD for the excellent rigorous review of our preliminary study. We believe that we have answered to the best of our ability the questions raised and revised the manuscript thoroughly. We look forward to the outcome of review of our submission.

Yours sincerely

Myron	Christodoulides, on behalf of all authors.
