# Supplementary material for: Comparison of a recombinant endonuclease III protein and its synthetic peptides in ELISA for the diagnosis of tegumentary leishmaniasis using human serum and urine samples: A preliminary study
Source: PLoS Negl Trop Dis. 2026 Jun 15;20(6):e0014440. doi: 10.1371/journal.pntd.0014440 (PMC13293512; doi:10.1371/journal.pntd.0014440)
Supplement: Attachment — Submitted filename: secondReview PNTD-D-26-00207.docx [file pntd.0014440.s004.docx]

**Comment 1**

I consider this question adequately answered.

**Comment 2 (STARD)**

The inclusion of the study description as a single-center, phase I, case-control diagnostic accuracy study using archived samples improves methodological transparency.

However, I consider that the original concern related to the STARD guidelines was only partially addressed. I agree that some items of the guidelines may have limited applicability in an exploratory study based on archived samples and without prospective participant recruitment. Still, important information for assessing potential selection bias remains missing, including:

• the origin and total universe of samples available in the biobank;

• the existence (or not) of excluded samples and the respective reasons;

• the justification for the number of samples used;

Although a complete STARD flowchart may not be strictly necessary in this context, a more detailed description of the sample selection process would substantially strengthen the manuscript.

**Comment 3 (Blinding)**

I consider this question adequately answered.

The revised version of the manuscript clarifies that the samples were previously coded and analyzed blindly and also reports the performance of independent experiments by different operators. This information increased the reliability of the results and addresses the concern originally raised.

**Comment 4 (Confirmation by PCR)**

I consider this question adequately answered.

The authors clarified that all patients with cutaneous and mucosal leishmaniasis tested positive for Leishmania braziliensis by PCR, also providing sufficient methodological details about the procedure used. This clarification improves the characterization of the reference standard employed in the study.

**Comment 5 (Extraordinary diagnostic performance)**

I consider this question adequately answered.

The revised version more appropriately emphasizes the preliminary nature of the findings and acknowledges that the high sensitivity, specificity, and AUC values observed should be confirmed in independent and more representative cohorts. I consider this concern satisfactorily addressed.

**Comment 6 (Longitudinal cohort and therapeutic monitoring)**

I consider this question adequately answered.

The revised version adequately acknowledges that:

• only a small number of patients were followed;

• only one post-treatment time point was evaluated;

• no comparative studies involving patients without therapeutic response were performed;

• it was not possible to correlate the reduction of antibodies with parasitological parameters.

These modifications make the interpretation of the results more balanced.

**Comment 7 (Antigen Stability)**

I consider this question adequately answered.

Although the authors present plausible arguments about the expected stability of recombinant proteins and synthetic peptides when properly produced and stored, I continue to consider that the absence of formal stability studies limits the interpretation of the follow-up results.

However, the current version of the manuscript was cautious in this regard, and the potential use of rENDO as a therapeutic monitoring marker is presented as preliminary and hypothesis-generating, which should be further investigated.

Comment 8 (Comparison with the rK39 test)

I consider this question adequately answered.

The revised version adequately characterizes the comparison with the Kalazar Detect™ test as exploratory and acknowledges the limitations inherent in using a test originally developed for visceral leishmaniasis. I consider this issue satisfactorily addressed.

**Conclusions**

The revised version presents more balanced conclusions, explicitly acknowledging the limitations related to the case-control design, the use of archived samples, the small sample size, and the absence of external validation.

I believe that the conclusions now more appropriately reflect the preliminary and exploratory nature of the study and are more aligned with the data presented.

**Reviewer's final opinion**

The revised version of the manuscript presents substantial improvements over the original version. Most methodological concerns have been adequately addressed, particularly regarding the characterization of the study, blinding procedures, diagnostic confirmation of cases, and more cautious interpretation of the results.

Only minor concerns remain regarding the transparency of the selection process for the archived samples, as well as the LM samples used in the follow-up study. These points can be resolved with minor additional revisions to the manuscript.
