# Supplementary material for: Comparison of a recombinant endonuclease III protein and its synthetic peptides in ELISA for the diagnosis of tegumentary leishmaniasis using human serum and urine samples: A preliminary study
Source: PLoS Negl Trop Dis. 2026 Jun 15;20(6):e0014440. doi: 10.1371/journal.pntd.0014440 (PMC13293512; doi:10.1371/journal.pntd.0014440)
Supplement: Peer review history

# Peer review: Decision Letter 0

**DOI:** 10.1371/journal.pntd.0014440.r001

**Authors:** Camila de Oliveira, Susan Madison-Antenucci

5 Mar 2026

PNTD-D-26-00207

Comparison of a recombinant endonuclease III protein and its synthetic peptides in ELISA for the diagnosis of tegumentary leishmaniasis using human serum and urine samples

PLOS Neglected Tropical Diseases

Dear Dr. Christodoulides,

Thank you for submitting your manuscript to PLOS Neglected Tropical Diseases. After careful consideration, we feel that it has merit but does not fully meet PLOS Neglected Tropical Diseases's publication criteria as it currently stands. Therefore, we invite you to submit a revised version of the manuscript that addresses the points raised during the review process.

Please submit your revised manuscript within by May 04 2026 11:59PM. If you will need more time than this to complete your revisions, please reply to this message or contact the journal office at plosntds@plos.org. When you're ready to submit your revision, log on to https://www.editorialmanager.com/pntd/ and select the 'Submissions Needing Revision' folder to locate your manuscript file.

Please include the following items when submitting your revised manuscript:

* A letter that responds to each point raised by the editor and reviewer(s). You should upload this letter as a separate file labeled 'Response to Reviewers'. This file does not need to include responses to any formatting updates and technical items listed in the 'Journal Requirements' section below.

* A marked-up copy of your manuscript that highlights changes made to the original version. You should upload this as a separate file labeled 'Revised Manuscript with Track Changes'.

* An unmarked version of your revised paper without tracked changes. You should upload this as a separate file labeled 'Manuscript'.

If you would like to make changes to your financial disclosure, competing interests statement, or data availability statement, please make these updates within the submission form at the time of resubmission. Guidelines for resubmitting your figure files are available below the reviewer comments at the end of this letter.

We look forward to receiving your revised manuscript.

Kind regards,

Camila I. de Oliveira

Academic Editor

PLOS Neglected Tropical Diseases

Susan Madison-Antenucci

Section Editor

PLOS Neglected Tropical Diseases

Shaden Kamhawi

co-Editor-in-Chief

PLOS Neglected Tropical Diseases

orcid.org/0000-0003-4304-636XX

Paul Brindley

co-Editor-in-Chief

PLOS Neglected Tropical Diseases

orcid.org/0000-0003-1765-0002

**Additional Editor Comments:**

Reviewers have identified issues with the current version of the manuscript. We ask that you please address the questions raised and supply a modified version of the manuscript in which all changes made to the original version are highlighted. We also ask you to supply a point-by-point response to the questions raised for appreciation, together with the revised version. In this revised version, please address the limitations of the study, and conclusions should be stated accordingly. We appreciate your effort and look forward to receiving the modified version.

**Journal Requirements:**

If the reviewer comments include a recommendation to cite specific previously published works, please review and evaluate these publications to determine whether they are relevant and should be cited. There is no requirement to cite these works unless the editor has indicated otherwise.

Please ensure that the funders and grant numbers match between the Financial Disclosure field and the Funding Information tab in your submission form. Note that the funders must be provided in the same order in both places as well.

**Reviewers' Comments:**

Reviewer's Responses to Questions

**Key Review Criteria Required for Acceptance?**

As you describe the new analyses required for acceptance, please consider the following:

**Methods**

-Are the objectives of the study clearly articulated with a clear testable hypothesis stated?

-Is the study design appropriate to address the stated objectives?

-Is the population clearly described and appropriate for the hypothesis being tested?

-Is the sample size sufficient to ensure adequate power to address the hypothesis being tested?

-Were correct statistical analysis used to support conclusions?

-Are there concerns about ethical or regulatory requirements being met?

Reviewer #1: 1. The study followed a single-center, case-control, phase I diagnostic accuracy design using archived samples. However, this is not explicitly stated in the methods.

2. The manuscript does not include a participant flow diagram as recommended by STARD. Missing STARD elements include Flow diagram of patient inclusion; Description of missing or excluded samples; Justification of sample size; Description of blinding procedures; Handling of indeterminate results.

3. Please clarify whether the ELISA interpretation was performed blindly.

4. Although TL diagnosis was based on clinical evaluation combined with smear microscopy and PCR, the manuscript does not clearly state: Whether all cases were PCR-confirmed; Whether discrepant results occurred;

Reviewer #2: Major Issues:

1. Cross reactivity data

a. Was L. brazeiliensis the only species causing TL included in the study cohort? Were other TL causing species endemic to the area identified, recorded using PCR and included in the study? If so, the paper could benefit sharing the list of leishmania species identified to understand species dependent assay performance.

b. The data lacks in mentioning if the ELISA was able to distinguish between CL and ML in an unknown sample. Since CL and ML can be caused by different species of leishmania- is strain identification using this assay ideal to establish clinical treatment protocol post diagnosis?

c. Method section is missing the technique used to identify malaria cases in the cross-reactive diseases (CRD) group. Figure legend often misses to mention if the malaria cases were included in the analysis of the CRD group.

d. The method section mentions that Detect™ Rapid Test for Visceral Leishmaniasis was used (Later it is mentioned it was used to establish the data in table 3) but misses to mention if this assay was used for inclusion criteria- to confirm TL or exclude VL in the study cohort.

2. Additionally, it is unclear from table 3 if the HC and CRD samples were used to assess false-positive and true-negative.

3. Graph in Fig 5A detecting CL cases in serum is significantly below the cutoff, the figure legend attributes this to peptide 2 but results discussion attributes this data to SLA. Please clarify.

4. Please include the graphical data showing variability of the 15 ML patient samples used for antibody follow-up in treated patients’ portion of the study. The results describe the average IgG reduction in the samples but is missing the variability data.

5. It is difficult to confidently claim anti-rENDO antibodies can be used as a prognostic biomarker since the data does not include information on how the biomarker behaves in cases where patient doesn’t show clinical improvement or if the patient takes a different disease course. There is also lack of data showing correlation of these antibodies with a standard clinical evaluation e.g. PCR values detecting parasitic load vs anti-rENDO antibodies.

Minor issues

1. B-cell epitope selection:

a. B cell peptide chosen for the assay are from endonuclease III (ENDO) which is a cytoplasmic protein for leishmania parasite. It will be beneficial to include a short discussion of why a cytoplasmic protein instead of surface antigen was chosen for this ELISA assay and potential mechanism on how this protein may be presented to the host immune system for B-cell antibody development.

b. The paper could benefit from discussion of how conserve these linear B-cell epitopes are across multiple leishmania species as shown in the supplementary data and its implication in clinical treatment decision making post diagnosis.

2. SLA using for this study was isolated only from L. Brasiliensis, the paper lacks discussion of rationale for choosing only this ML causing species instead of multiple parallel TL causing species. Would the authors expect the assay performance to change based on SLA from other species?

3. The study could benefit from a sample stability testing- especially for urine samples due to their acidic nature, to see how long the antigen binding antibodies stays sable in the sample before analysis.

**********

**Results**

-Does the analysis presented match the analysis plan?

-Are the results clearly and completely presented?

-Are the figures (Tables, Images) of sufficient quality for clarity?

Reviewer #1: 1. The study reports:

100% sensitivity and specificity for rENDO (serum and urine)

100% sensitivity and specificity for Peptide 2

AUC = 1.0 in multiple ROC analyses

While promising, this perfect diagnostic separation is uncommon in tissue leishmaniasis studies, particularly for cutaneous leishmaniasis, where antibody titers are often low.

Results should be interpreted with caution and conclusions should be considered with moderation.

2. The follow-up cohort (n = 15 patients with mucosal leishmaniasis, LM) showed a reduction in antibody levels six months after treatment.

However, some important limitations should be considered.

First, the methodology does not clearly describe how this longitudinal comparison was performed.

Second, it is unclear whether the 15 LM samples correspond to a subset of the original 30 LM cases included in the diagnostic analysis or whether they represent a distinct group. If only half of the original LM cohort was included, the rationale for excluding the remaining samples should be explicitly stated.

Third, the sample size is small, which substantially limits the strength of any conclusions about post-treatment dynamics. Furthermore, only a single post-treatment time point (six months) was evaluated, preventing assessment of the kinetics of antibody reduction or possible fluctuations over time.

No protein and synthetic peptides stability or assay stability studies were reported. Therefore, it cannot be ruled out that the observed reduction in antibody detection may be partially attributed to decreased antigen stability or assay performance, rather than a true biological reduction in antibody levels. This concern is particularly relevant because the SLA-based assay continued to demonstrate persistent reactivity, suggesting that antibody responses may not decrease uniformly across antigens.

Finally, there is insufficient evidence to support the authors' hypothesis that these assays can serve as markers for treatment monitoring.

3. The manuscript compares rENDO performance with the Kalazar Detect™ Rapid Test, which is designed for visceral leishmaniasis (VL), not TL.

This comparison may not be methodologically appropriate and should be interpreted cautiously.

If retained, it should be described as exploratory.

Reviewer #2: The paper is missing a few clarifications from the methods describing the testing of disease in patient cohorts. Additionally, the paper is missing the graphical data for antibody follow-up following treatment portion of the study.

**********

**Conclusions**

-Are the conclusions supported by the data presented?

-Are the limitations of analysis clearly described?

-Do the authors discuss how these data can be helpful to advance our understanding of the topic under study?

-Is public health relevance addressed?

Reviewer #1: The study presents promising preliminary data suggesting that rENDO and derived synthetic peptides may have high diagnostic accuracy for tegumentary leishmaniasis using both serum and urine samples.

However, the extraordinary diagnostic performance reported must be interpreted with caution due to the case–control design, limited sample size, and absence of validation cohorts.

With improved methodological transparency, strengthened discussion of limitations, moderated conclusions, and closer adherence to STARD guidelines, the manuscript could make a valuable contribution to the field of TL diagnostics.

Reviewer #2: It is difficult to confidently claim anti-rENDO antibodies can be used as a prognostic biomarker since the data does not include information on how the biomarker behaves in cases where patient doesn’t show clinical improvement or if the patient takes a different disease course. There is also lack of data showing correlation of these antibodies with a standard clinical evaluation e.g. PCR values detecting parasitic load vs anti-rENDO antibodies.

**********

**Editorial and Data Presentation Modifications?**

Use this section for editorial suggestions as well as relatively minor modifications of existing data that would enhance clarity. If the only modifications needed are minor and/or editorial, you may wish to recommend “Minor Revision” or “Accept”.

Reviewer #1: Minor revision

Reviewer #2: (No Response)

**********

**Summary and General Comments**

Use this section to provide overall comments, discuss strengths/weaknesses of the study, novelty, significance, general execution and scholarship. You may also include additional comments for the author, including concerns about dual publication, research ethics, or publication ethics. If requesting major revision, please articulate the new experiments that are needed.

Reviewer #1: The manuscript presents a relevant and innovative diagnostic approach for tegumentary leishmaniasis (TL), evaluating a recombinant endonuclease III protein (rENDO) and two synthetic peptides in ELISA platforms using both serum and urine samples. The inclusion of urine as a non-invasive biological sample is a notable strength and adds translational value. However, the extraordinarily high diagnostic performance reported (100% sensitivity and specificity, AUC = 1.0 in multiple analyses) requires careful methodological clarification and a more cautious interpretation. The study has scientific merit but requires improvements in methodological transparency, statistical robustness, and alignment with diagnostic accuracy reporting standards.

Strengths:

Innovative non-invasive urine-based ELISA. Rational antigen selection based on immunoproteomics. Inclusion of cross-reactive disease panel. Evaluation of synthetic peptides. Preliminary longitudinal assessment.

Limitations:

Incomplete adherence to STARD reporting standards. The manuscript does not fully comply with all items recommended by the STARD checklist for reporting diagnostic accuracy studies, particularly regarding participant flow, blinding procedures, and detailed methodological transparency.

Single-centre design. The study was conducted at a single centre, which may limit the generalizability of the findings to other epidemiological settings or patient populations.

Small subgroup sample sizes.Although the overall sample size is reasonable for a phase I exploratory study, several subgroups are relatively small. This may reduce statistical robustness and increase uncertainty in subgroup-specific performance estimates.

Insufficient methodological detail. The study design, participant selection process, and reference standards for each diagnostic group are not described in sufficient detail. Greater transparency is required regarding inclusion criteria, diagnostic confirmation methods, and whether test interpretation was performed blinded to clinical classification.

Perfect AUC values with potential risk of overestimation.The reporting of AUC values equal to 1.0 raises concern about potential overestimation of diagnostic performance.

Reviewer #2: Authors use leishmania cytoplasmic protein- endonuclease III (ENDO), to develop a set of recombinant protein (rENDO) and pair of peptide antigens specific ELISA for diagnostic and monitoring using patient serum and urine samples in the context of tegumentary leishmaniasis (consisting of cutaneous leishmaniasis and mucosal leishmaniasis). Authors discuss the limitations of current gold standard for diagnosis dependent of parasitological test and establish the need for an immunological technique oriented and cost-effective diagnostic assay. Assay shows strong sensitivity and specificity metric for the recombinant protein and peptide antigen and outperforms soluble leishmania antigen (SLA) obtained from L. braziliensis strain. The assay also demonstrates chosen antigen's ability to selectively diagnose CL and ML over other cross-reactive diseases- malaria, chagas, HIV, and leprosy.

However, the diagnostic test lacks the ability to distinguish between leishmania species that can affect clinical treatment protocol that is usually followed after a positive diagnostic test. Additionally, the evidence shown in the paper do not support the claim of the potential use of anti-rENDO antibodies as a prognostic biomarker for TL.

**********

PLOS authors have the option to publish the peer review history of their article (what does this mean?). If published, this will include your full peer review and any attached files.

If you choose “no”, your identity will remain anonymous but your review may still be made public.

**Do you want your identity to be public for this peer review?**  For information about this choice, including consent withdrawal, please see our Privacy Policy.

Reviewer #1: No

Reviewer #2: No

[NOTE: If reviewer comments were submitted as an attachment file, they will be attached to this email and accessible via the submission site. Please log into your account, locate the manuscript record, and check for the action link "View Attachments". If this link does not appear, there are no attachment files.]

**Figure resubmission:**

While revising your submission, we strongly recommend that you use PLOS’s NAAS tool (https://ngplosjournals.pagemajik.ai/artanalysis) to test your figure files. NAAS can convert your figure files to the TIFF file type and meet basic requirements (such as print size, resolution), or provide you with a report on issues that do not meet our requirements and that NAAS cannot fix.

After uploading your figures to PLOS’s NAAS tool - https://ngplosjournals.pagemajik.ai/artanalysis, NAAS will process the files provided and display the results in the "Uploaded Files" section of the page as the processing is complete. If the uploaded figures meet our requirements (or NAAS is able to fix the files to meet our requirements), the figure will be marked as "fixed" above. If NAAS is unable to fix the files, a red "failed" label will appear above. When NAAS has confirmed that the figure files meet our requirements, please download the file via the download option, and include these NAAS processed figure files when submitting your revised manuscript.

**Reproducibility:**

To enhance the reproducibility of your results, we recommend that authors of applicable studies deposit laboratory protocols in protocols.io, where a protocol can be assigned its own identifier (DOI) such that it can be cited independently in the future. Additionally, PLOS ONE offers an option to publish peer-reviewed clinical study protocols. Read more information on sharing protocols at https://plos.org/protocols?utm_medium=editorial-email&utm_source=authorletters&utm_campaign=protocols

Attachment Submitted filename: 2026 02 25 PNTD-D-26-00207 reviewers attachment.pdf

# Author comment: Author response to Decision Letter 1

**DOI:** 10.1371/journal.pntd.0014440.r002

11 May 2026

Attachment Submitted filename: Detailed List of responses to the Reviewers.doc

# Peer review: Decision Letter 1

**DOI:** 10.1371/journal.pntd.0014440.r003

**Authors:** Camila de Oliveira, Susan Madison-Antenucci, Camila de Oliveira, Susan Madison-Antenucci

4 Jun 2026

Dear Dr. Christodoulides,

We are pleased to inform you that your manuscript 'Comparison of a recombinant endonuclease III protein and its synthetic peptides in ELISA for the diagnosis of tegumentary leishmaniasis using human serum and urine samples: a preliminary study' has been provisionally accepted for publication in PLOS Neglected Tropical Diseases.

Before your manuscript can be formally accepted you will need to complete some formatting changes, which you will receive in a follow up email. A member of our team will be in touch with a set of requests.

Please note that your manuscript will not be scheduled for publication until you have made the required changes, so a swift response is appreciated.

IMPORTANT: The editorial review process is now complete. PLOS will only permit corrections to spelling, formatting or significant scientific errors from this point onwards. Requests for major changes, or any which affect the scientific understanding of your work, will cause delays to the publication date of your manuscript.

Should you, your institution's press office or the journal office choose to press release your paper, you will automatically be opted out of early publication. We ask that you notify us now if you or your institution is planning to press release the article. All press must be co-ordinated with PLOS.

Thank you again for supporting Open Access publishing; we are looking forward to publishing your work in PLOS Neglected Tropical Diseases.

Best regards,

Camila I. de Oliveira

Academic Editor

PLOS Neglected Tropical Diseases

Susan Madison-Antenucci

Section Editor

PLOS Neglected Tropical Diseases

Shaden Kamhawi

co-Editor-in-Chief

PLOS Neglected Tropical Diseases

orcid.org/0000-0003-4304-636XX

Paul Brindley

co-Editor-in-Chief

PLOS Neglected Tropical Diseases

orcid.org/0000-0003-1765-0002

***********************************************************

p.p1 {margin: 0.0px 0.0px 0.0px 0.0px; line-height: 16.0px; font: 14.0px Arial; color: #323333; -webkit-text-stroke: #323333}span.s1 {font-kerning: none

Reviewer's Responses to Questions

**Key Review Criteria Required for Acceptance?**

As you describe the new analyses required for acceptance, please consider the following:

**Methods**

-Are the objectives of the study clearly articulated with a clear testable hypothesis stated?

-Is the study design appropriate to address the stated objectives?

-Is the population clearly described and appropriate for the hypothesis being tested?

-Is the sample size sufficient to ensure adequate power to address the hypothesis being tested?

-Were correct statistical analysis used to support conclusions?

-Are there concerns about ethical or regulatory requirements being met?

Reviewer #1: I thank the authors for their careful revision of the manuscript and for the detailed responses provided. The revised version has substantially improved in terms of methodological transparency, discussion of limitations, and interpretation of the findings. Most of the concerns raised in the previous review have been adequately addressed.

Comment 1

I consider this issue adequately addressed.

Comment 2 (STARD reporting)

The inclusion of the study design as a single-center, case-control, phase I diagnostic accuracy study using archival samples improves the methodological transparency of the manuscript.

However, I believe that my original concern regarding STARD reporting has been only partially addressed. I agree that some STARD items may have limited applicability in an exploratory study based on archival samples without prospective participant recruitment. Nevertheless, important information relevant to the assessment of potential selection bias remains unclear, including:

the source population and the total number of samples available in the biobank;

whether any samples were excluded and, if so, the reasons for exclusion;

the rationale for the sample size included in the study.

Although a complete STARD flow diagram may not be strictly necessary in this context, a more detailed description of the sample selection process would substantially strengthen the manuscript.

Comment 3 (Blinding procedures)

I consider this issue adequately addressed.

The revised manuscript now clearly states that samples were coded before testing and analyzed in a blinded manner. In addition, independent ELISA experiments were performed by different operators. These clarifications improve confidence in the reported findings and satisfactorily address the original concern.

Comment 4 (PCR confirmation of cases)

I consider this issue adequately addressed.

The authors clarified that all cutaneous and mucosal leishmaniasis cases included in the study were PCR-positive for Leishmania braziliensis and provided sufficient methodological details regarding the molecular diagnostic procedure. This clarification strengthens the characterization of the reference standard used in the study.

Reviewer #2: (No Response)

**********

**Results**

-Does the analysis presented match the analysis plan?

-Are the results clearly and completely presented?

-Are the figures (Tables, Images) of sufficient quality for clarity?

Reviewer #1: Comment 5 (Extraordinary diagnostic performance)

I consider this issue adequately addressed.

The revised manuscript now appropriately emphasizes the preliminary nature of the findings and acknowledges that the exceptionally high sensitivity, specificity, and AUC values reported should be confirmed in larger and independent validation cohorts. The interpretation of these results is now more balanced and scientifically cautious.

Comment 6 (Longitudinal cohort and treatment monitoring)

I consider this issue adequately addressed.

The revised manuscript appropriately acknowledges that:

only a small number of patients were included in the follow-up analysis;

only a single post-treatment time point was evaluated;

no comparative analyses involving treatment failures were performed;

no correlation with parasitological parameters was available.

These additions substantially improve the interpretation of the longitudinal findings.

However, I encourage the authors to explicitly clarify whether the 15 mucosal leishmaniasis patients included in the follow-up analysis correspond to a subset of the original diagnostic cohort or to an independent group of patients.

Comment 7 (Antigen stability)

I consider this issue adequately addressed.

Although formal stability studies were not performed, the revised manuscript now discusses this limitation appropriately and presents the treatment-monitoring findings as preliminary and hypothesis-generating. This more cautious interpretation is appropriate given the available data.

Comment 8 (Comparison with rK39 test)

I consider this issue adequately addressed.

The revised manuscript now clearly frames the comparison with the Kalazar Detect™ test as exploratory and acknowledges the limitations associated with comparing a visceral leishmaniasis diagnostic assay with tegumentary leishmaniasis cases. This concern has been satisfactorily addressed.

Reviewer #2: (No Response)

**********

**Conclusions**

-Are the conclusions supported by the data presented?

-Are the limitations of analysis clearly described?

-Do the authors discuss how these data can be helpful to advance our understanding of the topic under study?

-Is public health relevance addressed?

Reviewer #1: The revised manuscript presents more balanced conclusions, explicitly acknowledging the limitations related to the case-control design, the use of archival samples, the relatively small sample size, and the absence of external validation cohorts.

I believe the conclusions are now more appropriately aligned with the data presented and with the exploratory nature of the study.

Reviewer #2: (No Response)

**********

**Editorial and Data Presentation Modifications?**

Use this section for editorial suggestions as well as relatively minor modifications of existing data that would enhance clarity. If the only modifications needed are minor and/or editorial, you may wish to recommend “Minor Revision” or “Accept”.

Reviewer #1: The revised manuscript represents a substantial improvement over the original submission. Most methodological and interpretative concerns have been adequately addressed, particularly regarding study characterization, blinding procedures, case confirmation, discussion of limitations, and moderation of the conclusions.

Only minor concerns remain regarding the transparency of the archival sample selection process and clarification of the mucosal leishmaniasis follow-up cohort. These issues could be resolved through minor revisions and do not require additional experimental work.

Reviewer #2: (No Response)

**********

**Summary and General Comments**

Use this section to provide overall comments, discuss strengths/weaknesses of the study, novelty, significance, general execution and scholarship. You may also include additional comments for the author, including concerns about dual publication, research ethics, or publication ethics. If requesting major revision, please articulate the new experiments that are needed.

Reviewer #1: This research presents high translational potential and innovative character, especially due to the proposed biomarkers applicable to urine samples. However, it is an exploratory phase I study, whose results should be interpreted with caution due to the case-control design, the use of archived samples, the limited sample size, and the absence of independent validation. The findings are promising and justify prospective multicenter studies to confirm the diagnostic and prognostic utility of the evaluated antigens.

Reviewer #2: The authors have made the changes and meaningful additions based on our reviewer comments in the previous review. No additional changes are recommended.

**********

PLOS authors have the option to publish the peer review history of their article (what does this mean?). If published, this will include your full peer review and any attached files.

If you choose “no”, your identity will remain anonymous but your review may still be made public.

**Do you want your identity to be public for this peer review?**  For information about this choice, including consent withdrawal, please see our Privacy Policy.

Reviewer #1: No

Reviewer #2: No

Attachment Submitted filename: secondReview PNTD-D-26-00207.docx

# Editor report: Acceptance letter

**DOI:** 10.1371/journal.pntd.0014440.r004

**Authors:** Camila de Oliveira, Susan Madison-Antenucci, Camila de Oliveira, Susan Madison-Antenucci

Dear Dr. Christodoulides,

We are delighted to inform you that your manuscript, "Comparison of a recombinant endonuclease III protein and its synthetic peptides in ELISA for the diagnosis of tegumentary leishmaniasis using human serum and urine samples: a preliminary study," has been formally accepted for publication in PLOS Neglected Tropical Diseases.

We have now passed your article onto the PLOS Production Department who will complete the rest of the publication process. All authors will receive a confirmation email upon publication.

The corresponding author will soon be receiving a typeset proof for review, to ensure errors have not been introduced during production. Please review the PDF proof of your manuscript carefully, as this is the last chance to correct any scientific or type-setting errors. Please note that major changes, or those which affect the scientific understanding of the work, will likely cause delays to the publication date of your manuscript. Note: Proofs for Front Matter articles (Editorial, Viewpoint, Symposium, Review, etc...) are generated on a different schedule and may not be made available as quickly.

Soon after your final files are uploaded, the early version of your manuscript will be published online unless you opted out of this process. The date of the early version will be your article's publication date. The final article will be published to the same URL, and all versions of the paper will be accessible to readers.

For Research Articles, you will receive an invoice from PLOS for your publication fee after your manuscript has reached the completed accept phase. If you receive an email requesting payment before acceptance or for any other service, this may be a phishing scheme. Learn how to identify phishing emails and protect your accounts at https://explore.plos.org/phishing.

Thank you again for supporting open-access publishing; we are looking forward to publishing your work in PLOS Neglected Tropical Diseases.

Best regards,

Shaden Kamhawi

co-Editor-in-Chief

PLOS Neglected Tropical Diseases

Paul Brindley

co-Editor-in-Chief

PLOS Neglected Tropical Diseases
